# *Furin*, a transcriptional target of NKX2-5, has an essential role in heart development and function

**DOI:** 10.1371/journal.pone.0212992

**Published:** 2019-03-06

**Authors:** Laurent Dupays, Norma Towers, Sophie Wood, Anna David, Daniel J. Stuckey, Timothy Mohun

**Affiliations:** 1 The Francis Crick Institute, London, United Kingdom; 2 Centre for Advanced Biomedical Imaging, University College London, London, United Kingdom; Imperial College London, UNITED KINGDOM

## Abstract

The homeodomain transcription factor NKX2-5 is known to be essential for both normal heart development and for heart function. But little is yet known about the identities of its downstream effectors or their function during differentiation of cardiac progenitor cells (CPCs). We have used transgenic analysis and CRISPR-mediated ablation to identify a cardiac enhancer of the *Furin* gene. The *Furin* gene, encoding a proprotein convertase, is directly repressed by NKX2-5. Deletion of *Furin* in CPCs is embryonic lethal, with mutant hearts showing a range of abnormalities in the outflow tract. Those defects are associated with a reduction in proliferation and premature differentiation of the CPCs. Deletion of *Furin* in differentiated cardiomyocytes results in viable adult mutant mice showing an elongation of the PR interval, a phenotype that is consistent with the phenotype of mice and human mutant for *Nkx2-5*. Our results show that *Furin* mediate some aspects of *Nkx2-5* function in the heart.

## Introduction

Morphogenesis of the four-chambered heart involves the transition between different differentiation states of cardiac progenitors and is regulated at the transcriptional level. *Nkx2-5* is high in the cardiac regulatory hierarchy and is expressed in all cardiac progenitors, including cells from the first (FHF) and anterior (AHF) heart fields [1, 2]. It has been shown to regulate the specification of cardiac progenitor cells (CPCs), the differentiation of cardiac chambers and to be necessary for the formation and maintenance of different elements of the cardiac conduction system [1, 3–7].

At the earliest stages of cardiac morphogenesis, *Nkx2-5*, acts along with *Bmp2* and phospho-Smad1 in a negative feedback loop to regulate the temporal balance between specification and differentiation of the cardiac progenitor pool [1]. Whilst the repressor effect of *Nkx2-5* on the *Bmp2*-phospho-Smad1 pathway appears to be critical in regulating the size of the CPC pool, the molecular mechanisms behind that interaction are poorly understood. In particular, it remains to be established if the effect of *Nkx2-5* in that negative feedback loop is direct or indirect.

The rhythmic and synchronised contractions of the atria and ventricles rely on the generation and conduction of the electrical activity through a specialised tissue: the cardiac conduction system (CCS) [8]. *Nkx2-5* is an essential player in both the formation and the functional maintenance of the physiological properties of the CCS [3–5].

*Furin* is a secretory proprotein convertase (PC) that activates precursor proteins into biological active forms by limited proteolysis at one or multiple internal sites [9]. The PCs are believed to be responsible for the maturation of a variety of protein precursors including growth factors, transmembrane receptors, endocrine hormones and adhesion molecules [9–12]. Multiple potential substrates of *Furin* have been implicated in different aspect of cardiac differentiation and development. For example, *Bmp4* is necessary for outflow tract (OFT) and atrioventricular (AV) septation and has been shown to be processed by FURIN [13, 14]. Moreover, mice carrying a mutation that disrupts ordered cleavage of proBMP4 to BMP4 show developmental abnormalities, including cardiovascular defects [15].

In this manuscript, we show that NKX2-5 directly represses cardiac *Furin* expression. Furthermore, we show that *Furin* is required for the maturation of the CPCs and for the correct formation of the AV junction where it is expressed. Finally, we show that this function is, at least in part, mediated by modulation of the BMP pathway.

## Materials and methods

### Animals

All mice were maintained at The Francis Crick Institute, according to the Home Office UK Animals (Scientific Procedures) Act 1986 under the Project Licence of Timothy Mohun. All animals were bred, born and weaned at room temperature and maintained on a 12-hour light-dark cycle. Mice were housed in groups of four and fed a standard diet (2018s, Envigo). Mice were anaesthetised with 1.5–2.0% isoflurane in 2 L/min oxygen and euthanized by isoflurane. Mice were handled by experienced experimenters to minimize any eventual distress. Genotyping of the transgenic mice lines *Furin* floxed, *Nkx2-5-IRES-Cre* and *Nkx2-5-gfp* has been described previously [1, 16].

### Transgenic mouse experiments

The 920bp M10 region was amplified by PCR using the primers FurM10_f (AACCCCAGAAATTTGCTCACAT) and FurM10_r (CCTGGAACTCGGGGCTTTAT) and cloned in a hsp68-LacZ reporter gene [17]. Transient transgenic mouse embryos were generated by the Procedural Services section at the Francis Crick Institute by standard pronuclear microinjection techniques.

### *Furin* enhancer M10 deletion

The *Furin*^*ΔM10*^ allele was generated using CRISPR/CAS9 technology [18]. Two guides RNAs, 5’-atgcggttgtgatctctcgg-3’ and 5’-gccaccggcaaaggcgtggt-3’ flanking the M10 enhancer were cloned in P459 vector and transcribed *in vitro* using MEGAshortscript T7 kit (Life Technologies). Following purification using the MEGAclear kit (Life Technologies) the two sgRNAs, and *in vitro*-transcribed Cas9 mRNA were co-injected into the cytoplasm of fertilized mouse oocytes using standard protocols. Nine independent founders F0 were identified to carry the deletion. Two of them were backcrossed to wildtype mice for at least three generations, and offspring were used for subsequent analysis. Genotyping was performed using 5’-GGCTTCCCTCCCAGTGTGC-3’ and 5’-TTGCCAAAGTAGCCAGAAAC-3’ and 5’-CTAGCCACCAAGGAAGGAT-3’ and 5’-CCGCTGGGCTAGGCAAATG-3’ primers.

### Electrocardiography

ECGs were recorded non-invasively in conscious adult mice using the ECGenie system (Mouse Specifics). Analysis of individual ECG signals was then performed using e-MOUSE physiologic waveform analysis software (Chu et al., 2001).

### Ultrasound experiments

Ultrasound measurements were performed on a Vevo 2100 system (VisualSonics, California, CA, USA) using an MS550D 30‐MHz transducer (VisualSonics) as described previously [19].

### Electrophoretic mobility shift assay

Recombinant proteins were synthesized using a coupled transcription-translation system (Promega TnT T7/SP6 kit) and incubated with 0.5 ng radiolabelled oligonucleotide probe in binding buffer (20mM Tris pH 7.6, 75mM KCl, 0.25mg/ml, 1mM DTT, 10% glycerol) at room temperature for 10 minutes. For competition experiments, excess of unlabelled oligonucleotide probes was added and incubated for 10 minutes prior to addition of radiolabelled oligonucleotide probes. Binding reactions were analysed on 6% 0.5X TBE polyacrylamide gels electrophoresed at 150V; gels were dried prior to detection of signal by autoradiography. Probes used were, M10-c: GATGACTGCCCTTATTTGACC and M10mut: GATGACTGggaggATTTGACC.

### *In situ* hybridization

Embryos were digested for 15 minutes with 10μg/ml proteinase K in PBST. Rinsed 5 minutes in 0.2%, glycine/PBST, before post-fixation for 20 minutes in 4% PFA. Embryos were pre-hybridized in hybridization mix without the probe for one hour at 70°C, and hybridized overnight at 70°C with a *Furin* RNA probe (synthesized from whole IMAGE clone). Embryos were washed 3 times 30 minutes at 65°C in solution 1 (50% formamide, 5x SSC, and 0.1% Tween-20) and 3 times 30 minutes at 65°C in solution 2 (50% formamide, 2x SSC). Embryos were pre-blocked for 2 hours in 10% sheep serum in TBST before being incubated overnight at -4°C with an anti-DIG antibody (7500 dilution). Embryos were washed with TBST, 5 times 1 hour at room temperature, and revealed using NBT/BCIP reaction.

### Immunohistochemistry

Embryos were fixed in PFA 4% for 30 minutes and embedded in gelatine before cryosectioning. Sections were washed in PBS-0.01% Triton, 3 times, and incubated for an hour in PBS-10% Donkey serum-0.1% Triton. Sections were incubated overnight at -4°C with the primary antibody in the same buffer as described previously [20]. Sections were washed 3 times, 15 minutes in PBS-0.01%, and incubated an hour with the secondary antibody. After incubation, sections were washed, and mounted in Dako (Agilent). Antibodies used were: NKX2-5 (Santa Cruz), H3 (Upstate), TBX3 (Santa Cruz), HCN4 (abcam), ISL1 (Hybridoma bank), H3 (Upstate), cTNI (Abcam) and phospho-Smad1,5 (1:200; Cell Signalling).

### High-Resolution Episcopic Microscopy (HREM) imaging

Before embedding and imaging, samples were dehydrated then infiltrated with a JB-4 dye mix (Polysciences). Once embedded, samples were sequentially imaged using an HREM microtome with optics and camera. 3D models were generated using 3D volume rendering (Osirix, Pixmeo) as described previously [21].

### Dual luciferase reporter assay

The 920bp M10 region was cloned into the pGL3 promoter vector (Promega). M10 mutated enhancer was obtain using QuickChange II site directed mutagenesis kit (Agilent Technologies). Transient transfections were performed in HL-1 cells as described previously [22]. Results were normalised to a renilla transfection control.

### Quantitative RT-PCR analysis

mRNAs were extracted from dissected hearts and limbs using TRIzol (Thermo Fisher). cDNAs were prepared using the Superscript II amplification system (Invitrogen). PCRs were performed using standard conditions [22] with the primers: Furin mRNA (f_Furin CCAGAGCGCCCTTTGAAA, r_Furin CCAGTCGCAAGATAAAAAAATACTTTG), Actb (f_Actb CGGGACCTGACAGACTACCTC, r_Actb AACCGCTCGTTGCCAATA), Blm (f_Blm TGCGCTGCTTGGTGAAGA, r_Blm TCAAGGGAGAAATGACAATTGTGA), LacZ (r_LacZ ATCAGGATATGTGGCGGATGA, r_LacZ TGATTTGTGTAGTCGGTTTATGCA).

### Western blot

Body wall from E9.5 embryos were pooled by genotype and homogenized in 0.5 ml of RIPA buffer (20 mM Tris-HCl, pH 8.0; 150 mM NaCl; 0.1% SDS; 1% NP-40; 0.5% Sodium deoxycholate; and complete protease inhibitors cocktail; Roche). Separation, transfer and blotting were described previously [22]. The antibodies used were the mouse BMP4 (R&D system, MAB757, 1:500) and rabbit phospho-Smad1/5/8 polyclonal (1:1,000; Cell Signaling).

### Chromatin immunoprecipitation assay

Chromatin was prepared from 40 to 50 mouse embryonic hearts at stage E9.5. Embryos were dissected, AHF and heart were separately collected and processed. Pooled samples were fixed for 3 hours at room temperature in buffer (50mM HEPES pH7.9, 1mM EDTA, 1mM EGTA, 100mM NaCl, 0.07% butryric acid) containing 1.8% formaldehyde. Heart tissue was homogenised using an Ultra-Turrax, T25 basic (IKA-Werke), and pelleted at low speed. Samples were washed twice with ice-cold PBS with freshly added EDTA-free protease inhibitors (PI) (Roche). Samples were resuspended in 1.5ml lysis buffer (25mM Tris pH 7.5, 150mM NaCl, 1% Triton X100, 1% SDS, 2.5mM Sodium deoxycholate, PI) and transferred to RNase-free non-stick microfuge tubes (Ambion). Samples sonicated for 15 x 30 seconds on ice using a Branson Digital Sonicator with a 2.5mm stepped probe tip. Samples were spun at 15K using a bench top centrifuge for 15 minutes at 4oC. The supernatant was then pre-blocked with protein A/G sepharose (Perbio), pre-treated with BSA (Biorad) and Poly (dI-dC)-poly (dI-dC) (GE Healthcare). A proportion of the sample was kept at this stage as an input control. 150ml of chromatin was diluted 10-fold in ChIP dilution buffer (16.7mM Tris pH7.5, 0.01% SDS, 1.1% Triton, 1.2mM EDTA, 167mM NaCl) and 4mg of the antibody was added and incubated overnight at 4°C with rotation. The antibody:protein:DNA complexes were captured using pre-blocked protein A/G sepharose for 2 hours rotating at 4°C. The beads were then spun down and washed twice in wash buffer A (10mM HEPES pH 7.6, 1mM EDTA, 0.5mM EGTA, 0.25% Triton X100) and twice with wash buffer B (10mM HEPES pH7.6, 200mM NaCl, 1mM EDTA, 0.5mM EGTA, 0.01% Triton X100. After extensive washing the beads were resuspended in TE and cross-links reversed overnight at 65^°^C. Samples were adjusted to 0.1% SDS, digested with 10mg Proteinase K (Roche) at 50^°^C for 3 hours and extracted twice with phenol/chloroform. Samples were ethanol precipitated with glycogen carrier (Ambion), and resuspended in 100ml TE. Antibodies used in the ChIP assays were NKX-2.5 (N-19) sc-8697 (Santa Cruz) and MEIS1 (Abcam, ab19867). Three independent preparations of extracts were used for the real time PCR experiments.

### Quantitative real-time PCR analysis

The fold-enrichment of the M10 Furin region in the immunoprecipitated DNA was determined by Real-Time quantitative PCR (qPCR) using the ABI PRISM 7000 sequence detection system (Applied Biosystems). The region was considered enriched in binding if they displayed greater than a cut-off value of 1.5-fold. Primers were designed using Primer Express software (Applied Biosystems). Relative fold-enrichment was determined by normalising the Ct values for input (ΔCt) by subtracting the average Ct value of input from average Ct value of IP (ΔCt = averageCt_ChIP_- averageCt_input_). Enrichment was then calculated relative to a negative control (promoter region of eye specific g-crystallin gene) using the formula 2-(ΔCt_[M10]_-ΔCt_[gCrystallin]_). Fold enrichment was calculated by dividing the enrichment values from the antibody-specific- ChIP by the enrichment values obtained in the background (no antibody) control samples. Each experiment of PCR was performed in triplicate.

## Results

### NKX2-5 repress *Furin* cardiac expression

**Fig 1:**
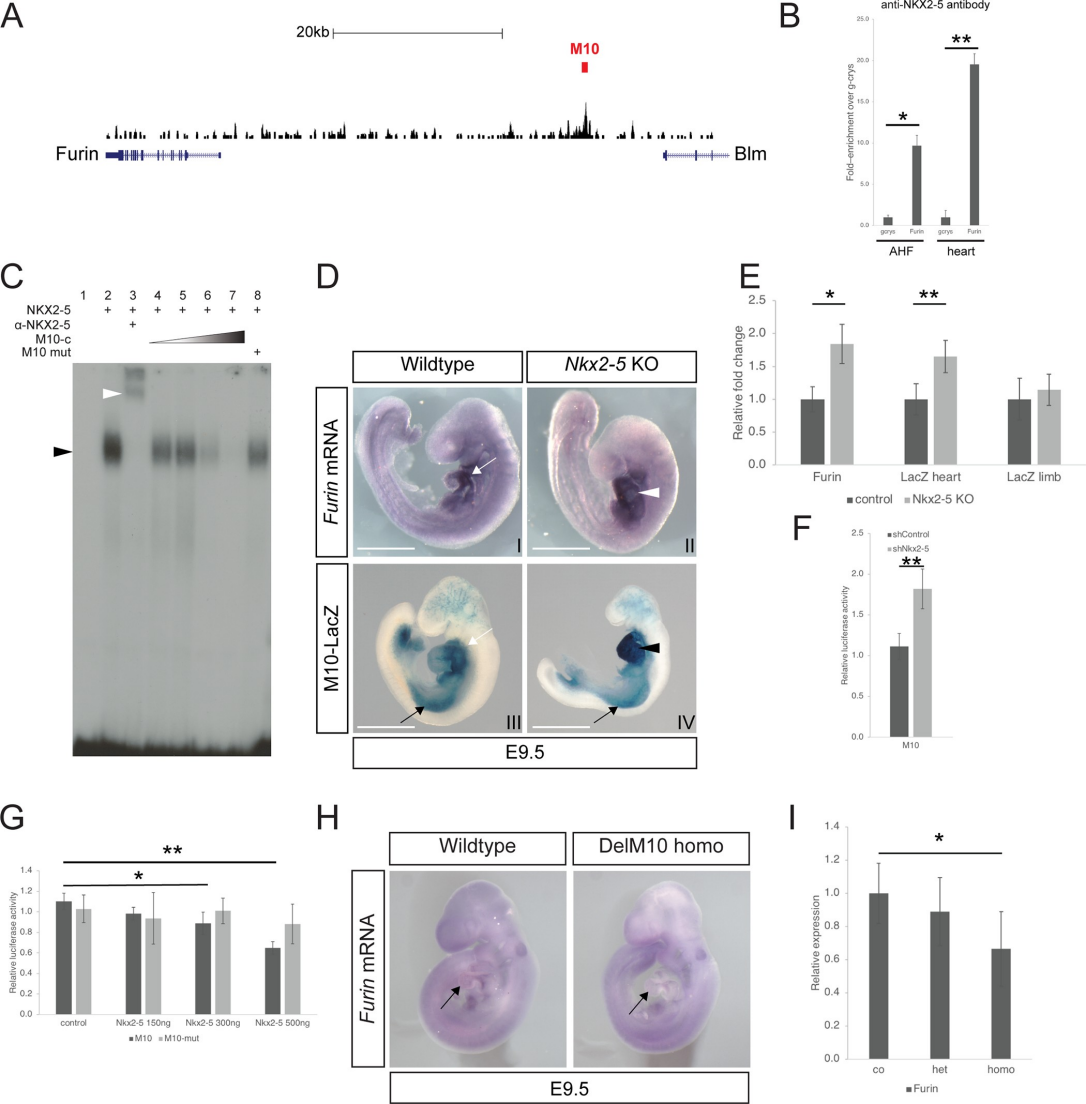
The enhancer of *Furin* M10 is repressed by NKX2-5. (A) ChIP-seq trace at the *Furin* locus. The region identified as enriched for NKX2-5 binding (M10) and subsequently tested in transgenic studies is indicated (red bar). Note the 3’ terminus part of the *Blm* gene. 20kb scale is shown. (B) ChIP analysis with an anti-NKX2-5 antibody, using chromatin purified from E9.5 AHF and heart. Three independent preparations of chromatin extracts were used for the real time PCR analysis with primers against the M10 region. For relative enrichment, a region of the gamma-crystallin gene (*gcrys*) was used as a negative control. (C) Electrophoretic mobility shift assay (EMSA) showing that NKX2-5 binds its motif (see methods) in the M10 probe (black arrowhead). Inclusion of a specific antibody against NKX2-5 results in a supershift of the band (white arrowhead, lane 3). Presence of unlabelled probe oligonucleotide results in specific competition (M10-c, lane 4 to lane 7). No competition is observed with a mutated unlabelled probe (lane 8). (D) Expression of *Furin* mRNA as detected using *in situ* hybridisation in wildtype and *Nkx2-5* knock out mouse embryo at E9.5. Note the increase staining for *Furin* in the heart of the mutants (white arrowhead). The transgene M10 recapitulates *Furin* expression in the heart (compare white arrows). Note the increase staining for the transgene in the heart of the mutants (black arrowhead) while the expression in the limbs remains comparable to wild type (compare black arrows). Scale bar 1mm. (E) Quantification of *Furin* and *LacZ* mRNA in heart and limb using qPCR in wildtype (n = 4) and *Nkx2-5* knock out embryos (n = 4). *Furin* and *LacZ* mRNA expression is increased by 1.84 and 1.64-fold respectively in mutants while *LacZ* expression remains similar in the limbs. (F) Relative luciferase activity in control and NKX2-5 deficient HL-1 cells using the M10-pGL3 fragment. Graph represents 3 different experiments. (G) M10-pGL3 Luciferase reporter activity decreases with cotransfection of increasing amounts of an *Nkx2-5* expressing vector. In contrast, luciferase activity from the M10-mut-pGL3 reporter vector is unaffected by *Nkx2-5* overexpression in HL-1 cells. (Only significant changes are indicated. Graph represents 3 independent transfections). (H) Whole mount *in situ* hybridization showing a reduction in *Furin* mRNA expression in the heart of a E9.5 mutant mouse embryo carrying a homozygous deletion of the enhancer M10 (compare black arrows). (I) *Furin* mRNA. expression is significantly reduced in the hearts of homozygous mutants’ embryos (n = 4) for M10 deletion. *: p<0.05, **: p<0.001 according to a two-tailed Student’s t-test.

Using data from our previous ChIPseq study [22, 23], we identified a 920 base pair region (referred as M10) which is 30kb upstream of the *Furin* transcription start site and enriched for NKX2-5 binding (Fig 1A). To confirm NKX2-5 binding on M10 we performed ChIP experiments using chromatin from samples prepared from the anterior heart field (AHF) and heart of embryonic day 9 (E9.5) embryos (Fig 1B). Using chromatin prepared from these tissues [22] (Fig 1B), we found that NKX2-5 bound specifically to the M10 region and the extent of binding was similar for both AHF and heart.

A subset of target genes for NKX2-5 were previously found to be bound by the transcription factor MEIS1. However, we could not find any significant enrichment for MEIS1 binding on M10 compared to the negative control (S1A Fig). We used an electrophoretic shift assay (EMSA) to confirm that NKX2-5 is able to bind *in vitro* to the sequence (TGCCCTTATTT), a motif present in M10 and identified in the sequence of the ChIPseq dataset (Fig 1C and S1B Fig). Again, MEIS1 was unable to bind to this motif *in vitro* (S1C Fig).

As described previously, we found that at E9.5 expression of murine *Furin* mRNA is strong in the heart and the lateral plate mesoderm while diffuse in the remainder of the embryo [24] Fig 1DI). Detailed section analysis showed that *Furin* was expressed in the AHF (S1D Fig, black arrowhead), the primitive atria and the outflow tract (S1D Fig, white arrowheads) and to a lesser extend in the primitive ventricle. *Furin* was also found in the endocardium and the proepicardium (S1D Fig). As development progressed, *Furin* expression became restricted to the central CCS (see the description below). To assess the nature of the regulation of *Furin* by *Nkx2-5* we looked at its expression in a knockout background. We found that *Furin* expression is upregulated in *Nkx2-5* mouse knockout embryos at E9.5 using *in situ* hybridisation (Fig 1DII). Similar results were obtained using quantitative RT-PCR (Fig 1E, see below). This suggests that *Nkx2-5* represses expression of *Furin*.

The M10 region was tested for its ability to drive cardiac expression in the developing mouse heart, using transient transgenic embryos. Three out of five embryos positive for the transgene expressed the LacZ reporter gene in the heart and limb (see expression of *M10-LacZ*, Fig 1D). The remaining wo transgenic embryos showed no expression of the transgene. A stable line for the *M10-LacZ* transgene was generated and transgene expression compared with endogenous *Furin* expression at two stages of development. This analysis showed that the M10 region drives cardiac expression in a similar way to the endogenous *Furin* (Figs 1D and 4 and S1D Fig). Moreover, on a *Nkx2-5* null mutant background, the transgene was found to be upregulated in the embryonic heart, in a similar manner to the endogenous *Furin* gene. This indicates that the enhancer is responsive to the level of NKX2-5 (Fig 1DIV). We used quantitative RT-PCR to measure the increase in *Furin* expression in the *Nkx2-5* null mutant embryos. *Furin* mRNA expression is 1.84 higher in the *Nkx2-5* null background, while *LacZ* mRNA from the reporter transgene is upregulated by 1.65-fold in the heart (Fig 1E). However, *LacZ* mRNA levels remained unchanged in the limb bud (Fig 1E). Expression level of the *Blm* gene, flanking the M10 region (Fig 1A), was not affected in the *Nkx2-5* knock-out (S1E Fig).

We examined the repressor function of NKX2-5 using an HL-1 cardiac cell line previously shown to have significantly reduced levels of NKX2-5 protein [22]. We tested the effect of NKX2-5 knockdown on the expression of a luciferase reporter carrying the M10 fragment (Fig 1F). Luciferase activity was significantly increased in HL-1 cells deficient for NKX2-5 compared with controls (M10; Fig 1F). Moreover, co-transfection with increasing amounts of an NKX2-5 expression vector progressively suppressed luciferase reporter activity. However, it had no effect after mutation of the *Nkx2-5* DNA binding site (Fig 1G).

To test directly whether the M10 region regulated the expression of *Furin* we deleted this fragment *in vivo*, using CRISPR/CAS9 technology. Two sgRNAs flanking the enhancer were transcribed *in vitro* and co-injected with CAS9 mRNA into the cytoplasm of fertilized mouse oocytes. We obtained 9 independent founder mice for the M10 deletion (described as *Furin*^*M10*Δ*/+*^; S1F Fig), which were identified using PCR (S1G and S1H Fig). Two independent mice lines carrying the deletion were outcrossed to wildtype mouse in order to eliminate non-specific mutations. *Furin*^*M10*Δ*/+*^ were then intercrossed to generate *Furin*^*M10*Δ*/M10*Δ^ embryos. At E9.5, embryos homozygous for the deletion (*Furin*^*M10*Δ*/M10*Δ^) we detected a weak decrease in expression of the *Furin* mRNA in the heart, as revealed by whole mount *in situ* hybridization (Fig 1H). We found a significant decrease in *Furin* expression in homozygous mutants using quantitative PCR (Fig 1I). In contrast, heterozygous embryos (*Furin*^*M10*Δ*/+*^) showed no significant change in *Furin* expression. This suggested that M10 was necessary for the correct expression level of *Furin* expression *in vivo* and was a *bona fide* enhancer of *Furin in vivo*. However, embryos homozygous for the M10 deletion showed no obvious phenotype at this early stage and we were able to recover homozygous adult mice for that genotype. This indicates that the modest reduction in *Furin* expression resulting from loss of M10 was insufficient to produce an obvious deleterious phenotype. Nevertheless, taken together, our data show that NKX2-5 directly represses *Furin* expression in the heart.

### *Furin* is necessary for heart development

**Fig 2:**
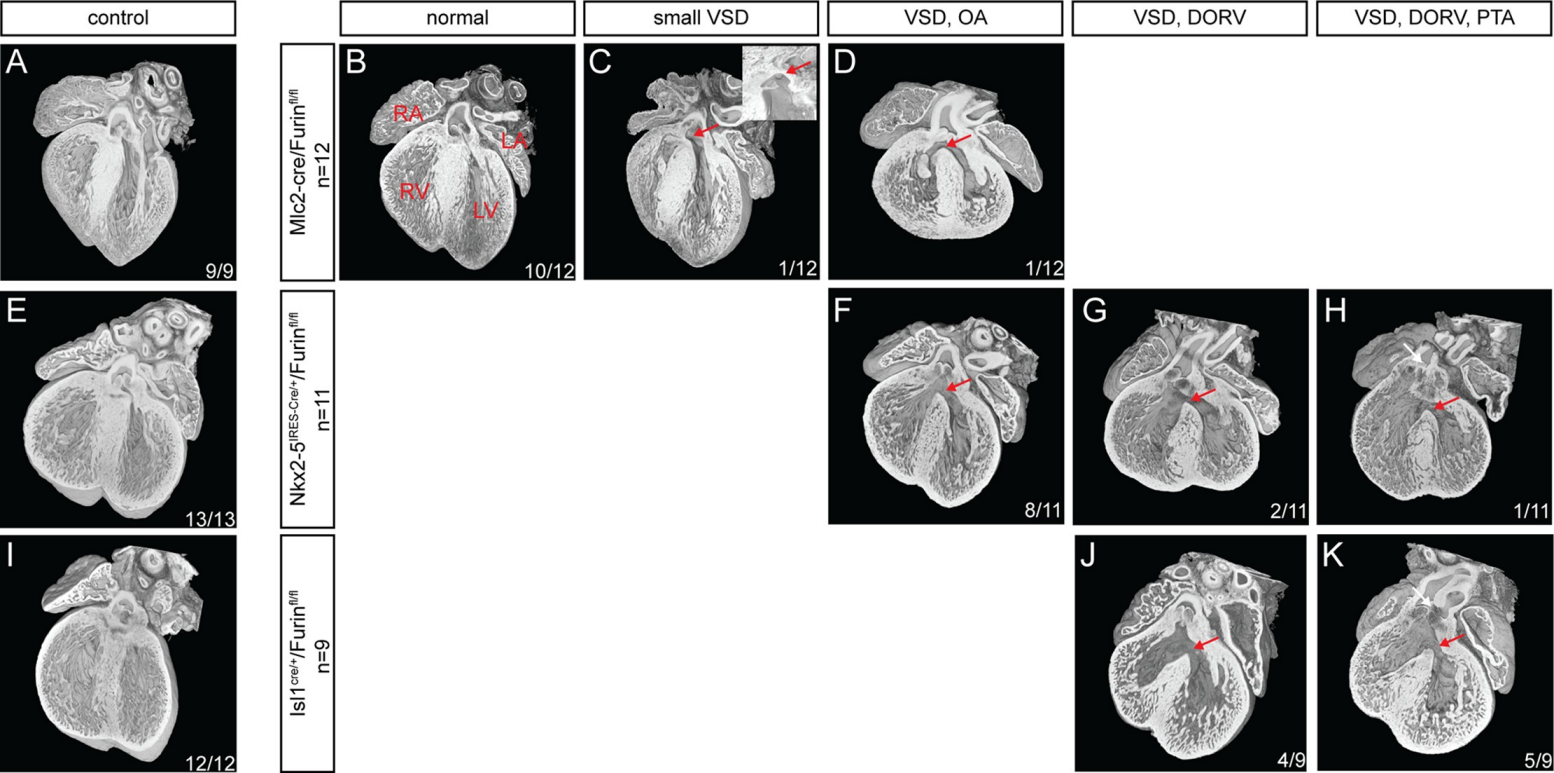
Characterisation of the cardiac phenotype in *Furin* mutants at E14.5. Four-chambered view of wild type controls (A, E and I) and mutant (B-D, F-H and J-K) *Furin* hearts for the different cre lines used in that study using high-resolution episcopic microscopy (HREM). Inset in C represent a left septal view of the VSD in the same image). Red arrows, VSD. White arrows, PTA. RA, right atria. LA, left atria. LV, left ventricle. RV, right ventricle, PTA, persistent truncus arteriosus. In each image is indicated: number of hearts with the phenotype/number of samples analysed.

Since *Furin* is a direct transcriptional target of NKX2-5, its activity may mediate some aspects of NKX2-5 function in the heart. To assess the function of *Furin* in cardiomyocytes we took advantage of a conditional line knock out for the *Furin* gene (*Furin*^*fl/fl*^) [16] and backcrossed it with a series of cardiomyocyte specific lines: *Isl1*^*cre/+*^ [25], *Nkx2-5*^*IRES-Cre/+*^ [26] and *MLC2-cre* [27]. While *Isl1*^*cre/+*^ and *Nkx2-5*^*IRES-Cre/+*^ lines allow recombination in cardiac progenitors, recombination with the *Mlc2-cre* line only occurs in differentiated cardiomyocytes (S2 Fig). Whilst we were able to retrieve *Mlc2-cre/Furin*^*fl/fl*^ mutant mice, no *Isl1*^*cre/+*^*/Furin*^*fl/fl*^ and *Nkx2-5*^*IRES-Cre/+*^*/Furin*^*fl/fl*^ weaning mutant mice could be recovered, suggesting embryonic lethality in each case. We proceeded to carry a morphological analysis of the different lines at E14.5 (Fig 2).

At E14.5 only two of the twelve *Mlc2-cre/Furin*^*fl/fl*^ mutant hearts analysed showed a morphological defect (Fig 2C and 2D). One embryo presented a small ventricular septal defect (VSD, red arrow in Fig 2C and inset) the other a larger VSD with an overriding aorta (red arrow in Fig 2D), the ten remaining were undistinguishable from wild type controls (Fig 2A and 2B). In contrast, at the same stage, all mutant hearts (*Isl1*^*cre/+*^*/Furin*^*fl/fl*^ and *Nkx2-5*^*IRES-Cre/+*^*/Furin*^*fl/fl*^) displayed a VSD and a malposition of the outflow tract (OFT) vessels (Figs 2F–2H, 2J and 2K).

Interestingly the cardiac defects we detected comprised a spectrum of malformations. Whilst most *Nkx2-5*^*IRES-Cre/+*^*/Furin*^*fl/fl*^ mutant hearts (72%, 8 out of 11) showed a VSD with an overriding aorta (OA), 18% (2 out of 11) showed a VSD with double outlet right ventricle (DORV) and 10% (1 out of 11) also showed a common proximal infundibulum resembling a persistent truncus arteriosus (PTA). For *Isl1*^*cre/+*^*/Furin*^*fl/fl*^ mutant hearts, approximatively half (44%, 4 out of 9) showed a VSD with DORV and half (56%, 5 out of 9) DORV and PTA. The difference in phenotype severity observed between *Isl1*^*cre/+*^ and *Nkx2-5*^*IRES-Cre/+*^ driver lines may be the consequence of a difference in expression in the CPCs. Indeed, whilst both lines are expressed in the CPCs, *Isl1*^*cre/+*^ appears to show a qualitatively stronger recombination in CPCs than *Nkx2-5*^*IRES-Cre/+*^ (S2 Fig).

These results indicate that *Furin* is required for cardiac development, specifically for the correct formation of the OFT. The absence of consistent morphological defects in *Mlc2-cre/Furin*^*fl/fl*^ mutant suggests that *Furin* is primarily required in the CPCs, cells in which the the *MLC2-cre* line is not active.

### *Furin* is required for normal maturation of CPCs

**Fig 3:**
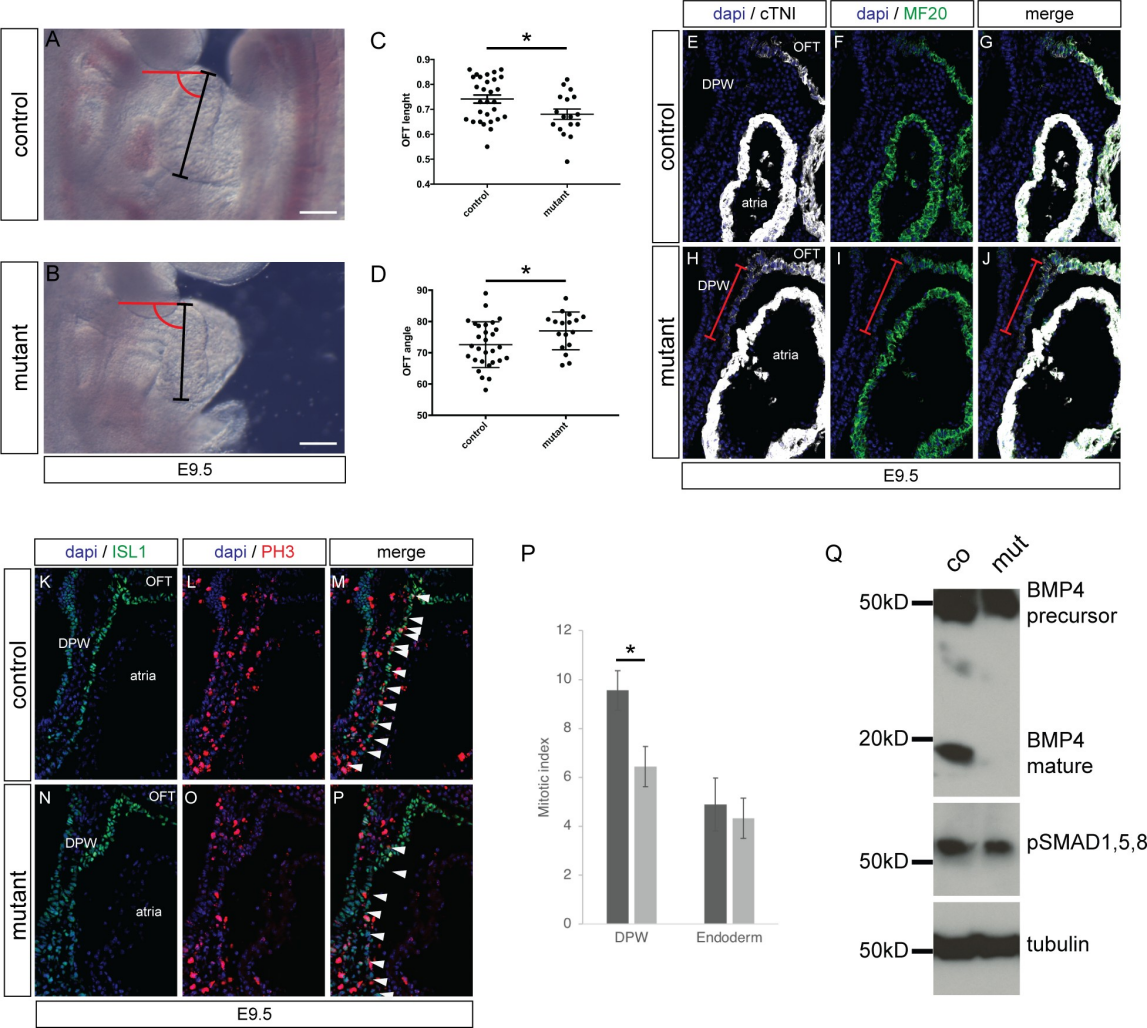
Defect in OFT formation of the *Furin* mutants. (A, B) Right side views of E9.5 control and *Nkx2-5*^*IRES-Cre/+*^*/Furin*^*fl/fl*^ mutant embryos. (C) Measurement of OFT length as shown in A and B with black bars. (D) Measurement of OFT angle as shown in A and B in red (n = 29 for control embryos and 17 for *Furin* mutants). *P<0.05 for unpaired Student’s t test. (E-F) Sagittal section showing ectopic expression of cTNI and MF20 in the dorsal pericardial wall (DPW) of mutant and control E9.5 embryos. Red brackets indicate the extend of the differentiated cells. Location of atria and OFT is shown. (K-P) Sagittal section showing PH3-positive cells in the DPW of control and mutant embryos. ISL1 staining identify CPCs. (P) Quantification showing a reduction of mitotic index in mutant embryos compared to control while it is unchanged in the underlying endoderm (n = 3 for each genotype). (Q) Western blot analysis of BMP4 and pSmad1,5,8 expression in protein extract from 3 pooled body walls of E9.5 control and mutant embryos.

To confirm that *Furin* is required at an early stage of cardiac development we examined embryos at an earlier stage for morphological defects. At E9.5, the structure of the OFT was assessed by measuring its length and angle (black line and red arc respectively in Fig 3A and 3B). This analysis showed that *Nkx2-5*^*IRES-Cre/+*^*/Furin*^*fl/fl*^ mutants embryos had a significantly shorter OFT with a larger angle between the proximal and distal OFT compared with control embryos (Fig 3A–3D). Similar results were found for *Isl1*^*cre/+*^*/Furin*^*fl/fl*^ mutant embryos (S2D–S2G Fig).

In order to identify the molecular changes associated with the alteration of OFT morphology, we examined expression of CPC differentiation markers. Cardiac troponin I (cTNI) and MF20 were both ectopically expressed in the AHF of the *Isl1*^*cre/+*^*/Furin*^*fl/fl*^ embryos at E9.5 (Red brackets in Fig 3E–3J). We also assessed proliferation in the AHF (as identified by ISL1 expression) and observed a significant reduction of phosphorylated histone H3 (PH3) positive cells (Fig 3K–3P). Taken together those results indicate that aberrant morphology of the OFT results from a decrease in proliferation of cardiac progenitors in the AHF and their ectopic differentiation.

To evaluate whether the defects in the AHF of *Furin* mutants could be the consequence of a decrease in BMP signalling, we looked for a change in BMP4 maturation. Using western blot analysis of protein extracted from E9.5 body wall from controls and mutants, we found a strong decrease in the presence of the mature BMP4 form compared with its BMP4 precursor (Fig 3Q). In agreement, the phosphorylation levels of Smad1,5,8 were reduced (Fig 3Q). Moreover, using immunohistochemistry, we found that the number of phospho-smad1,5 / ISL1 positive cells in the AHF of the mutants was significantly reduced compare to controls (S3G Fig), while the number of phospho-smad1,5 positive cells present in the atrial endocardium of both genotypes remained similar (S3H Fig). Taken together, these results suggest that a decrease in BMP signalling could be responsible for ectopic differentiation of CPCs.

### *Furin* enhancer M10 is a marker of the central conduction system

**Fig 4:**
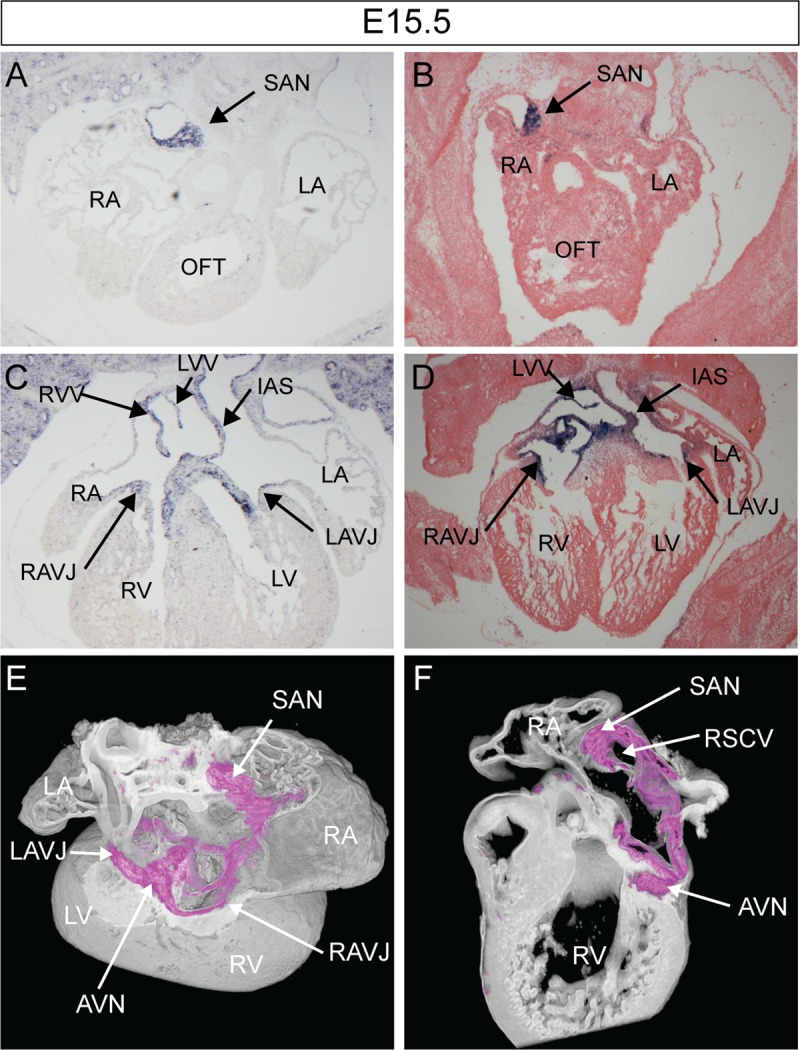
*Furin* is a marker of the central conduction system. (A-F) Study of *Furin* expression in the E15.5 mouse heart. (A, C) *Furin* mRNA expression as detected by *in situ* hybridisation. (B, D) M10 enhancer LacZ expression as detected after X-gal staining. (E, F) 3D rendering of β-galactosidase expression (purple) using dual-wavelength HREM [21][23]. (E) Cropped dorsal view to reveal the AV rings. (F) Sagittal view of the sliced heart at the level of the nodes. AVN, atrioventricular node; AVB, atrioventricular bundle; SAN, sinoatrial node; RAVR, right atrioventricular ring bundle; LA, RA, left/right atrium; L/RV, left/right ventricle; RSCV, right superior caval vein; VS, ventricular septum; RVV, right venous valve.

A more detailed analysis of the *M10-LacZ* transgenic line using dual-wavelength HREM imaging [21] showed that not only did the transgene recapitulate the endogenous *Furin* expression pattern at E9.5; at later stages it also mirrored endogenous expression in the sinoatrial node (SAN), atrioventricular node (AVN), atrioventricular bundle as well as the right and left atrioventricular junction (AVJ) (Fig 4 and S1D Fig). TBX3 is a well-known marker of the central conduction system [28] while HCN4 delineates the SAN [29]. Colocalisation of the β-galactosidase, expressed by the *M10-LacZ* line, with TBX3 and HCN4, confirmed that the transgene was expressed in the central CCS (S4 Fig) and suggests a role for *Furin* in that tissue. At E15.5, *Furin* was expressed in the AV cushions while the transgene was not (Fig 4C and 4D) indicating that the M10 enhancer drives most, but not all, of the endogenous expression of *Furin* in the embryonic heart.

### Atrioventricular conduction defects in *Furin* mutants

**Fig 5:**
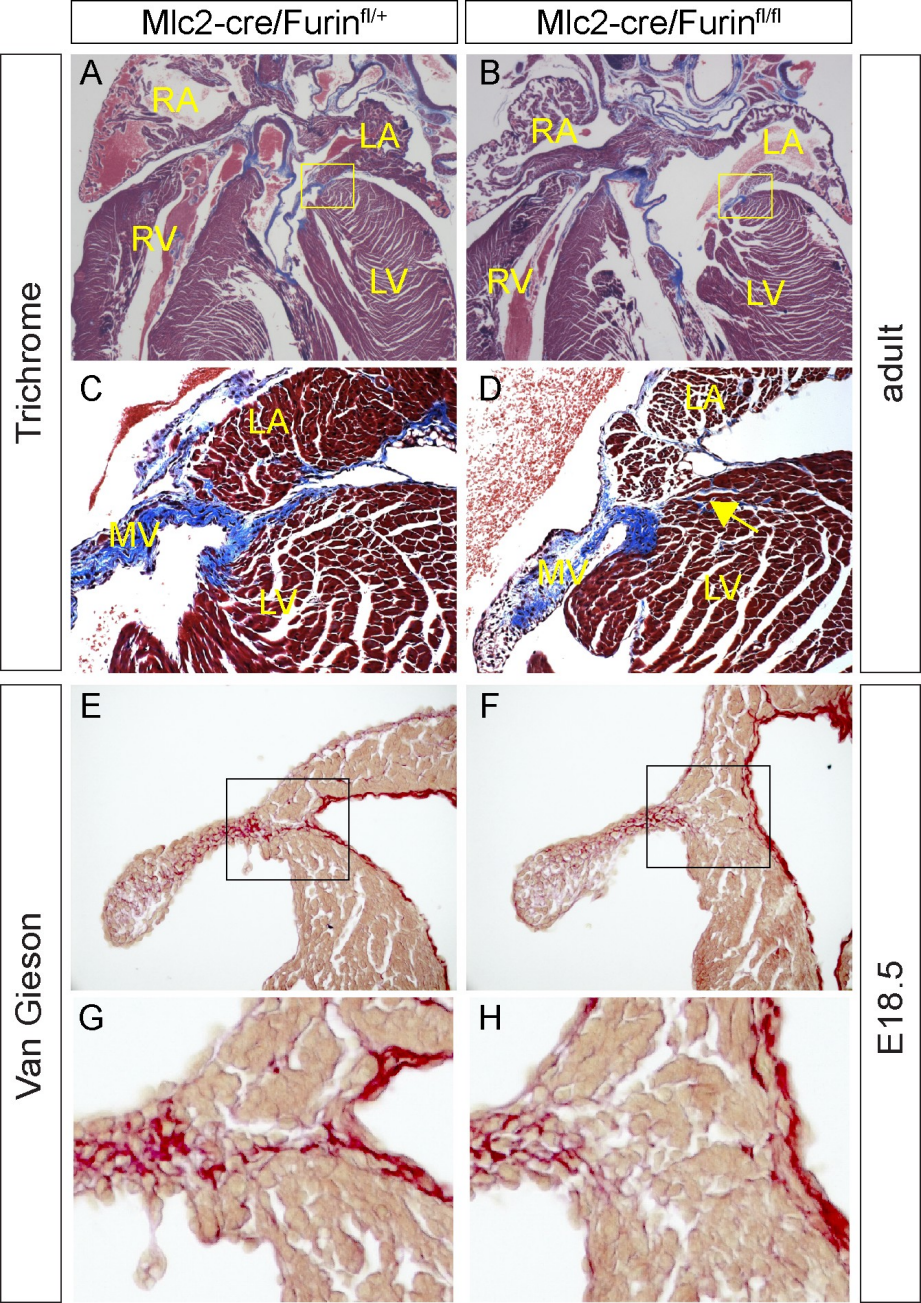
*Furin* mutant hearts present a defect of the AV junction. (A, D) Sections of an adult mouse wild type (n = 3) and mutant (n = 3) hearts (11 weeks old) stained with Masson-trichrome protocol to reveal connective tissue in blue. C and D show higher magnification images of the areas within the yellow squares in A and B. Note the myocardial continuity between the cardiomyocytes of the atria and the ventricle in mutants (yellow arrow in D). MV, mitral valve. (E-F) Sections of E18.5 mouse wild type (n = 2) and mutant (n = 2) hearts stained with Van-Gieson protocol to reveal connective tissue in red. G and H show higher magnification images of the areas within the black squares in E and F.

**Fig 6:**
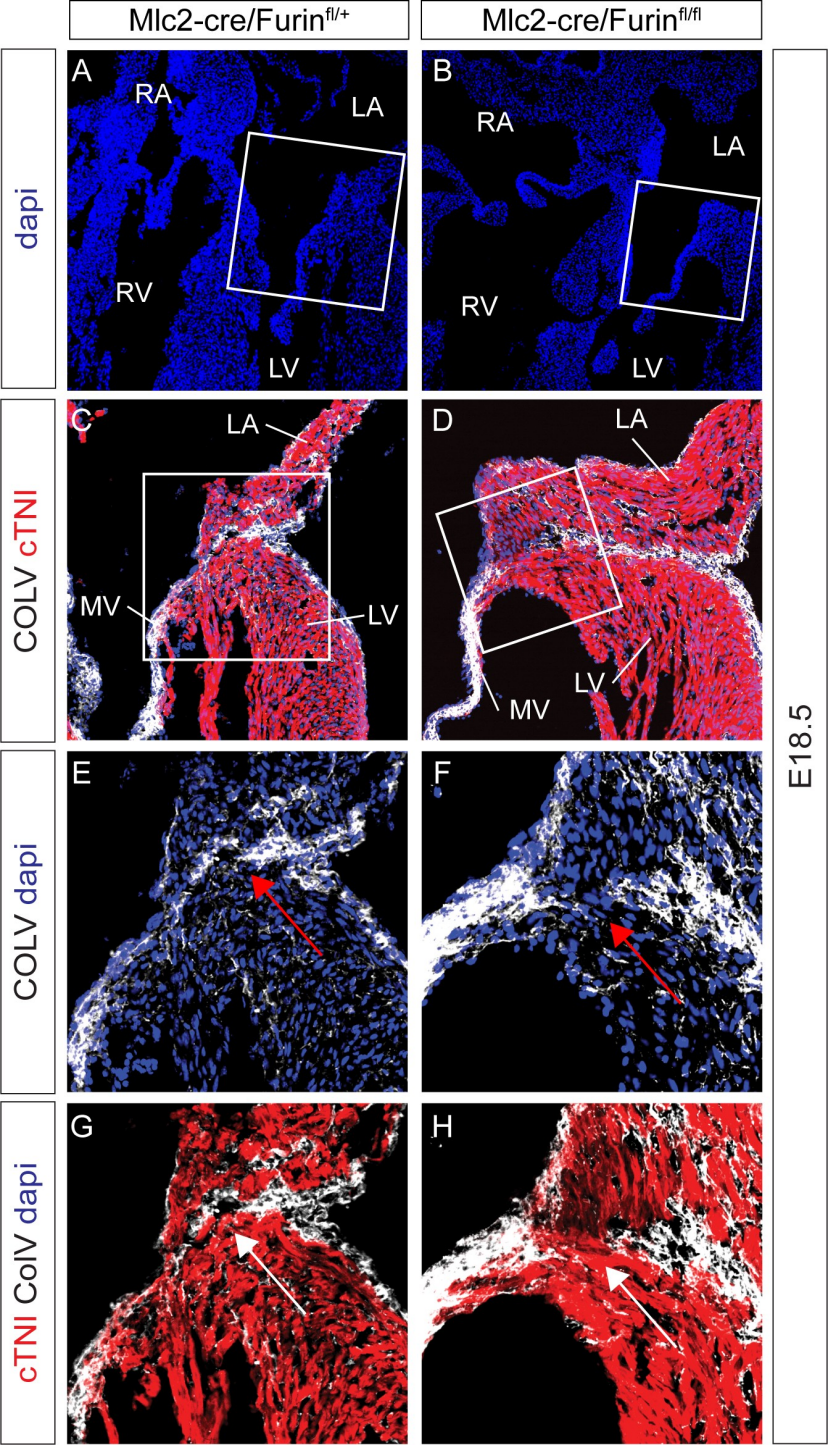
*Furin* mutant hearts present in COLLAGENV of the AV junction. (A-H) Immunofluorescences of E18.5 mouse wild type (n = 2) and mutant (n = 2) hearts revealing the defect in COLLAGENV deposition at the AV junction (Compare red arrows in E and F; white arrows in G and H). G and H show higher magnification images of the areas within the white squares in A and B. (E-H) high magnifications of squared area in C and D.

Consistent with the low penetrance of the morphological heart defects detected in *Mlc2-cre/Furin*^*fl/fl*^ mutant embryos, we recovered 86% of the adult mutant mice expected (18 mice mutants at weaning observed; 21 expected). We could not find any significant difference between control and mutant hearts in size or morphology of the SAN and AVN, as judged using the markers TBX3 and HCN4. However, histological analysis of the adult mutant heart identified a defect in the AV junction (Figs 5 and 6). Masson’s trichrome staining, which reveals connective tissue, revealed a defect in collagen deposition on the left AV junction of the adult heart, resulting in a continuity between the muscular part of the atria and ventricles (Fig 5C and 5D). This phenotype could be detected as early as E18.5 using van Gieson staining (Fig 5E–5H). Among the isoforms of collagen expressed in the heart, Collagen V has previously been shown to be a marker of the annulus fibrosus [30]. Deposition of Collagen V at the left AV junction was found to be defective in *Mlc2-cre/Furin*^*fl/fl*^ mutants (Fig 6C–6H), suggesting that *Furin* is necessary for the maturation of the annulus fibrosus on this side of the heart.

**Fig 7:**
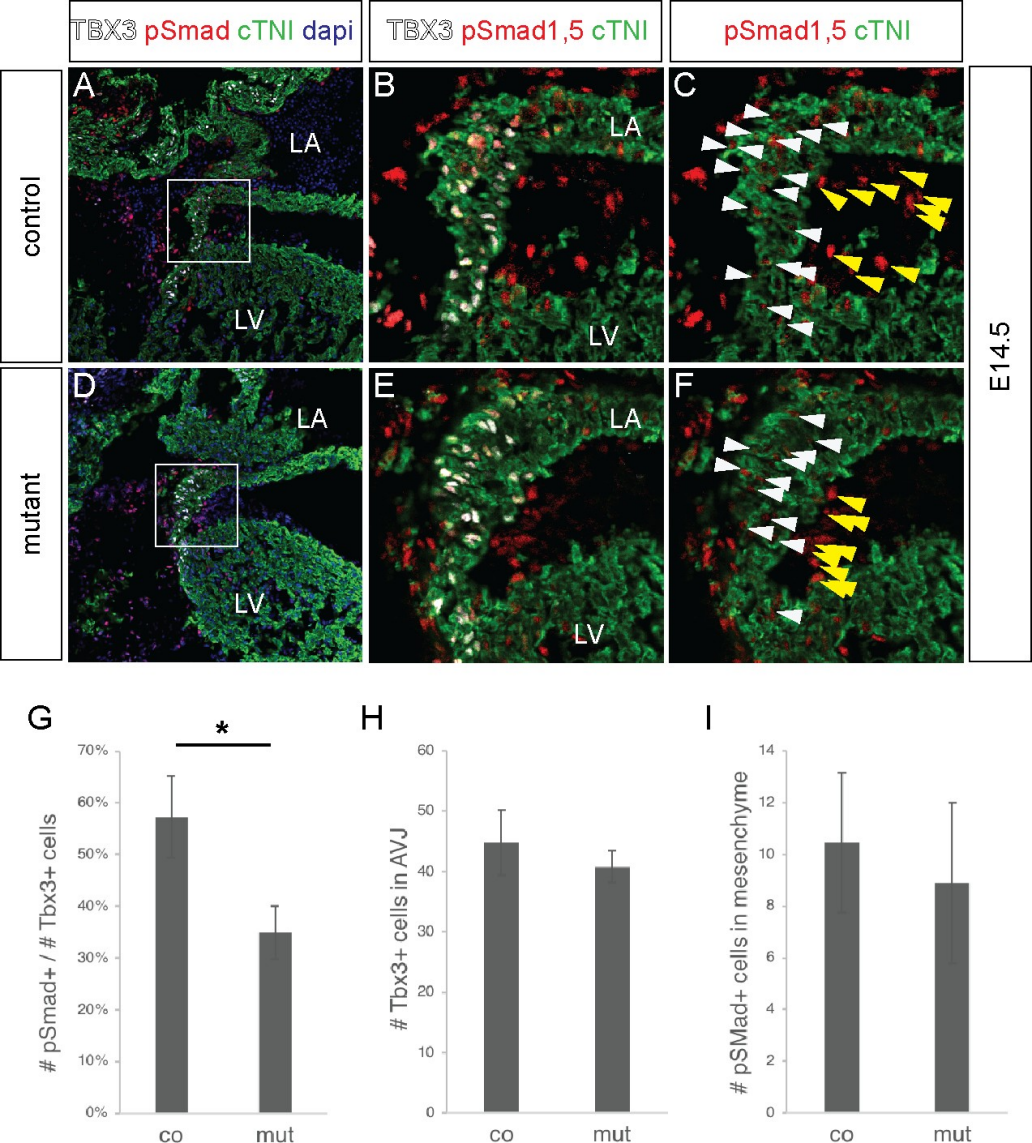
*Furin* mutant hearts show a decreased in the number of phospo-Smad positive cells at the AV junction. (A-F) Immunostaining showing the expression of pSMAD1/5 (red), cTNI (green) and TBX3 (white) in the left atrioventricular junction of a E14.5 mouse heart. B, C, E, F show high magnifications of the areas squared in A and D. White arrowheads indicate phospho-SMAD1/5 positive / Tbx3 positive cells. Yellow arrowheads indicate phospho-SMAD1/5 positive epicardial derived mesenchymal cells of the AV sulcus. (G) Graph showing a significant reduction in the number of phospho-SMAD1/5 positive cells in the TBX3 positive population of the AV canal. (n = 3 hearts analysed for each genotype). *P<0.05 for unpaired Student’s t test. (H) Graph showing that the number of TBX3 positive cells in the AV junction is equivalent in wildtype and control. (I) Graph showing that the number of phospho-SMAD1/5 positive cells in the epicardial derived mesenchymal cells of the AV sulcus is equivalent in wildtype and control.

The BMP pathway has previously been shown to be essential in the formation of the annulus fibrosus [31, 32]. Using immunohistochemistry, we found that the ratio of phospho-smad1,5/TBX3 positive cells was significantly reduced in the mutant AV junction compare to controls (Figs 7A–6G), while the number of TBX3 positive cells present in the AV junction of both genotypes remained similar (Fig 7H). The number of phospho-smad1,5 positive cells in the epicardial-derived mesenchymal cells of the AV sulcus was also similar between genotypes (Fig 7I).

**Fig 8:**
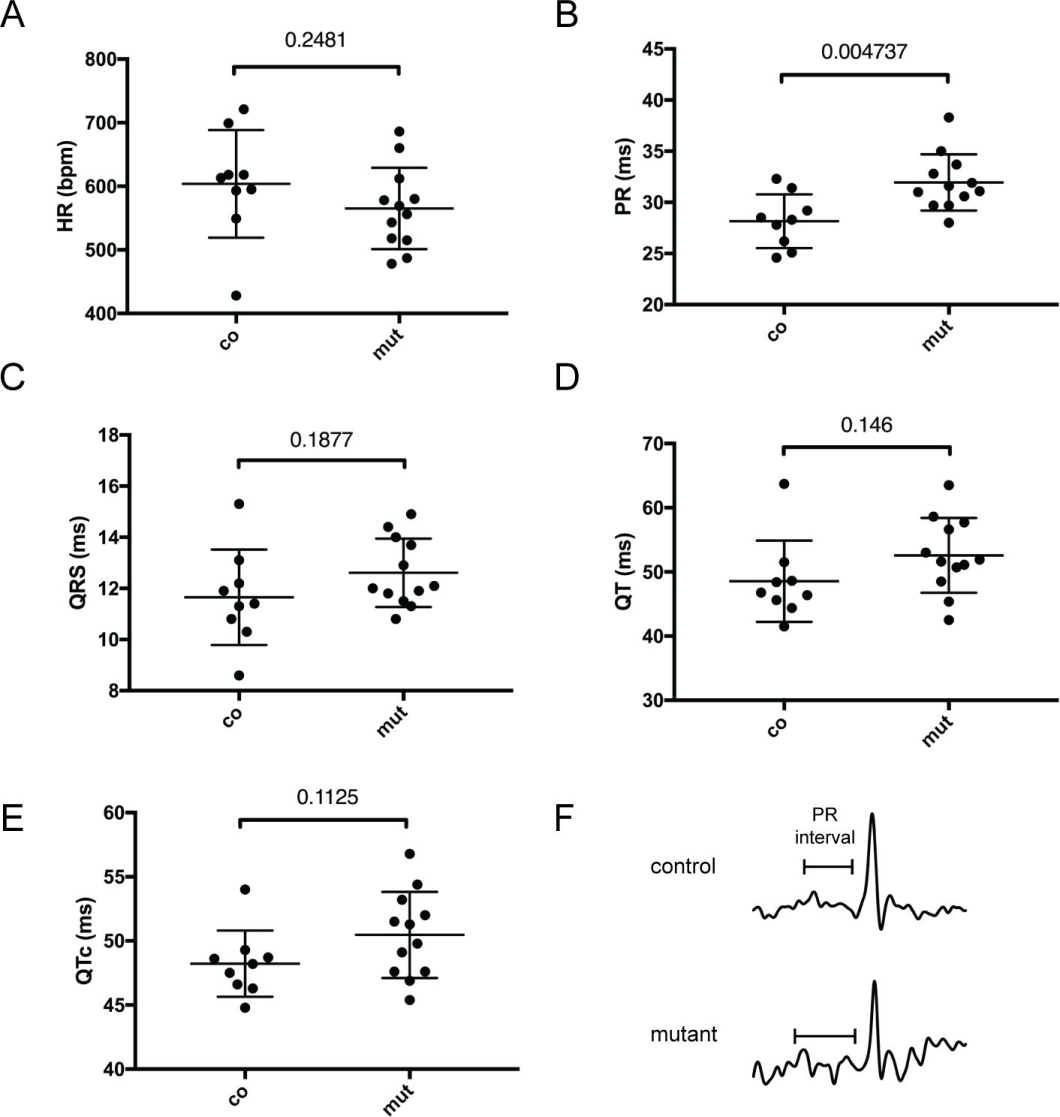
ECG characterisation of 11 weeks old *Furin* mutant mice. *Mlc2-cre/Furin*^*fl/fl*^ adult mice (mut, n = 12) show an increased PR interval (B) compared to control (co, n = 9) for a similar heart rate. (A). QRS (C), QT (D) and QTc (E) are not significantly different between genotypes. Means ± s.e.m. are shown. P values obtained from unpaired Student’s t test are indicated for each parameter.

These results suggest a defect in BMP signalling in the AV junction. Defects in this tissue are often associated with conduction defects therefore we investigated the electrophysiological proprieties of the mutant hearts. ECG recordings revealed that 3 month-old *Mlc2-cre/Furin*^*fl/fl*^ mutant mice presented a significant elongation of the PR interval (28.16 ms ± 0.8729, n = 9 in wildtype versus 31.95 ms ± 0.7916, n = 12 in mutants, Fig 8B) at a comparable heart rate to controls. The mutants were normal for other parameters (Fig 8). Ultrasound characterisation showed that the cardiac structure, function and flow of the *Mlc2-cre/Furin*^*fl/fl*^ mice at this age were nonetheless comparable to those of controls at the same age (S1 Table).

## Discussion

### *Furin* is a direct target of NKX2-5 repression

By studying the genome-wide distribution of binding by NKX2-5 to chromatin, we have identified one such region (M10) in close vicinity to the *Furin* gene. Transgenic analysis demonstrates that this region can drive expression of *LacZ* in the developing embryo in a pattern that largely matches endogenous expression of *Furin*. Comparable experiments against a *Nkx2-5* null mutant background show up-regulation of the *LacZ* transgender, a result similar to the up-regulation of endogenous *Furin* expression in the same genetic background. Furthermore, deletion of the enhancer *in vivo* results in down-regulation of *Furin* expression. Together, these findings demonstrate that M10 functions as a cardiac enhancer of *Furin*, mediating repression of *Furin* expression by NKX2-5. Since redundancy is a common feature of mammalian genomes [33], it is perhaps not surprising that the loss of this *Furin* enhancer was insufficient to completely abolish cardiac expression of this gene. We could not find any obvious morphological phenotype in the homozygous mutants for the M10 deletion (*Furin*^*M10*Δ*/M10*Δ^) at E9.5, and we could recover adult mice for that genotype. A more detailed analysis of these mutants will be carried out a future work to see if they present a defect in electrophysiological parameters comparable to that detected in adult *Mlc2-cre/Furin*^*fl/fl*^ mutants.

### *Furin* is necessary for cardiac progenitor’s differentiation

It was previously shown that embryos homozygous for *Furin* deletion cease developing before embryonic day 8 [24], such mutants showing abnormal morphogenesis of the yolk sac vasculature and a severe ventral closure defect. Such severe and early malformations precluded the analysis of the direct effect of *Furin* loss on cardiomyocyte formation or function [24]. While mice with a liver-specific deletion of *Furin* are viable [16], new-borns with an endothelial-specific deletion of *Furin* die shortly after birth and show cardiac malformations [34]. Moreover, FURIN activity is essential in extraembryonic and precardiac mesoderm [35].

In our study we show that embryos with a deletion of *Furin* in the CPCs die around mid-gestation, presenting a range of cardiac abnormalities, including VSD and defects in the OFT formation spanning OA, DORV and PTA. These findings demonstrate that *Furin* is required for normal cardiac development. Our data indicate that these defects are the consequence of premature differentiation and reduced proliferation of CPCs in the AHF, resulting in abnormalities in growth and morphogenesis of the OFT.

Such early differentiation defects affecting the CPCs have also been found in *Nkx2-5* mutants [1, 36]. *Nkx2-5* is part a negative feedback loop in which it inhibits BMP/Smad signalling [1]. BMP signalling itself coordinates the balance between proliferation and differentiation of CPCs [37]. Moreover, inactivation of *Bmp4* within the AHF-derived myocardium results in neonatal lethality, due to OFT defects [38]. BMP4 is a well-established substrate of FURIN, its maturation being required for OFT and AV septation [10, 14, 34, 39]. In agreement with this, disruption of BMP4 processing results in cardiovascular defects [15]. In our study, BMP4 processing was found to be strongly reduced, with the mature isoform of BMP4 nearly absent. Interestingly, phospho-smad levels were much less reduced reduced, suggesting that BMP4 is not the only BMP ligand acting in the CPCs. Indeed expression of other BMP’s in the AHF has previously been reported [40].

Taken together, our results suggest that *Nkx2-5* modulates BMP signalling by directly repressing *Furin* expression, thereby regulating the availability of active BMP4 necessary for the formation of the OFT.

### *Furin* is necessary for the maturation of the AV junction

In addition to this role in CPCs, using a cre-specific driver for differentiated cardiomyocytes (*Mlc2-cre*), we have identified an additional later role for *Furin* in cardiac function. Whilst *Mlc2-cre/Furin*^*fl/fl*^ adult mice are viable, they present an elongation of the PR interval, a phenotype that is consistent with the foetal expression of *Furin* in the central CCS and with the phenotype of mice and humans mutant for *Nkx2-5* [3, 41–43]. *Nkx2-5* has been shown to be an important player in both the formation and function of the CCS [3–5]. Human mutations of NKX2-5 cause a variety of heart malformations including: AV conduction abnormalities, AV block, atrial septal defect, VSD and Tetralogy of Fallot [2, 43, 44]. AV conduction abnormalities are one of the most common clinical representations of NKX2-5 mutations and can occur in the absence cardiac structural malformations. Accordingly, genome wide association study revealed an association between the genetic variations in NKX2-5 and the PR interval [45]. Moreover, mice carrying a loss of function allele for NKX2-5 show PR prolongation and heart failure without any apparent morphological defects [46].

If *Nkx2-5* is necessary for the maturation and function of the CCS, it remains unclear how a transcription factor expressed virtually in all cardiomyocytes can have such a restricted effect on a discrete specialised tissue. We hypothesise that *Furin*, which is expressed specifically in the central CCS mediates that role.

Furthermore, in *Mlc2-cre/Furin*^*fl/fl*^ mice we have found persistent myocardial strands that connected the left atrium with the left ventricle, concomitant with a defect in collagen deposition at the AV junction. This suggests a defect in the formation of the annulus fibrosus. We also found that this defect is associated with a decrease in BMP signalling. Defects in this tissue, which is necessary for the electrical insulation of the atria from the ventricles, lead to the formation of accessories pathways. This results in ventricular pre-excitation, a feature of Wolff-Parkinson-White syndrome [47, 48].

Several pathways have been shown to be necessary for the formation of the AV junction [47–49]. Conditional deletion of the ALk3/Bmpr1a receptor in the AV canal demonstrates a role of the AV myocardium in the development of the annulus fibrosus [31, 32], indicating a critical role for BMP signalling in its formation and development. Our result suggests a mechanism in which *Nkx2-5* modulates BMP signalling in the AV junction through the repression of *Furin*.

The *Furin* mutant showed an unexpectedly longer PR interval while previously described models with similar defect had a shorter PR interval or pre-excitation [31, 47]. The discrepancies observed could be explained by several factors. Our ECGs were recorded on younger live mice while older anesthetized mice were used in earlier studies [31, 47]. Moreover, the affected area in the left AV junction in our model is more lateral (S5 Fig) than the affected area in the hearts of Aanhaanen et al. (see Fig 6E in Aanhaanen et al.), suggesting that the location of the defect may have an impact on the ECG parameters. Furthemore, it is possible that other, as yet unidentified defects could be present either in our mutants or the mutants described previously, and these may have an impact on the electrophysiology of the heart.

## Supporting information

S1 Fig *Furin* is a direct target of NKX2-5. (A) ChIP analysis using chromatin purified from E9.5 AHF and heart and an anti-MEIS1 antibody. Note that there is no significant difference in enrichment of binding between *Furin* and a negative control region derived from the gamma-crystallin gene. Representative results from quantitative PCR using primers for the M10 region are presented. (B) Diagram showing the global conservation of the M10 sequence (920bp) between mouse (mm8) and human (hg18). The area in red (269bp in mouse) is highly conserved (sequence conservation superior at 70%). A base level sequence comparison is shown underneath. The position of the *Nkx2-5* DNA binding site analysed in that study is shown in red. (C) EMSA showing that MEIS1 doesn’t bind the M10 probe in vitro (Lanes 2 to lane 6) while it binds the Popdc2 enhancer identified previously (Lane 7, black arrowhead). (D) Sagittal sections of embryos at E9.5. White arrowheads indicate expression in the myocardium while black arrowheads indicate expression in the anterior heart field. PE, proepicardium. (E) Relative quantitative RT-PCR for Blm mRNA expression in isolated embryo hearts of control and Nkx2-5 knock-out (Nkx2-5-gfp/Nkx2-5-gfp) at E9.5. (F) Sequence of the allele deleted in the 9 stable lines generated (Line 671-Line 681). Lines 676 and Line 678 were kept for subsequent analysis. sgRNA sequences are labelled in red, PAM sequences in blue. (G) Diagram showing the strategy used for offsprings genotyping. The schematic position of the sgRNA used to ablate the M10 enhancer in the genome is indicated with black arrowheads. The internal (red arrowheads) and overlapping (blue arrowheads) primer pairs used for genotyping are shown. (H) PCR analysis of the DNA of three mice (wildtype, heterozygote and homozygote for the deletion). Primers used in each lane are indicated with coloured arrowheads. Note the absence of amplification with the red/internal primer in the homozygous line.
(TIF)

S2 Fig Characterisation of Cre-Driver mouse lines used in that study. **(A-C)** At E9.5 *Isl1*^*cre/+*^ and *Nkx2-5*^*IRES-Cre/+*^ lines are expressed in the AHF while *Mlc2-cre* line is not (Brackets). Expression of the reporter LacZ appears to be broader and stronger in the AHF of the Isl1-cre/R26R-LacZ embryos compare to Nkx2-5-IRES-cre/R26R-LacZ embryos (Compare brackets in B and C). (D, F) Right side views of E9.5 control and Isl1^cre/+/^Furin^fl/fl^ mutant embryos. (E) Measurement of OFT length as shown in D and F with black bars. (F) Measurement of OFT angle as shown in D and F in red (n = 12 for control embryos and 12 for *Furin* mutants). *P<0.05 for unpaired Student’s t test.
(TIF)

S3 Fig *Furin* mutant embryos show a decreased in the number of phospo-Smad positive cells in the AHF. (A-F) Immunostaining showing the expression of pSMAD1/5 (red) and ISL1 (green) in a wildtype (A, C and E) and mutant (B, D and F) embryo E9.5 mouse heart on sagittal sections. White arrowheads indicate phospho-SMAD1/5 positive / ISL1 positive cells in the AHF. Yellow arrowheads indicate phospho-SMAD1/5 positive endocardial cells of the atria. (G) Graph showing a significant reduction in the number of phospho-SMAD1/5 / ISL1 positive cells in the AHF. *P<0.05 for unpaired Student’s t test. (H) Graph showing that the number of phospho-SMAD1/5 positive cells in the endocardium of the atria in wildtype (n = 3) and control (n = 3) is not significantly changed.
(TIF)

S4 Fig Colocalization of the transgene M10 with TBX3. Expression of the transgene M10 is detected with an antibody against the ß-galactosidase. Panels show transversal section of a E17.5 mouse heart with a focus on the sinoatrial node (A-D), the tricuspid valve (E-H) and AVN (I-L). White arrows in D, H and L show that the transgene M10 is expressed in the sinoatrial node (D), the right atrioventricular junction (H) and the atrioventricular node (L). (M-P) Immunofluorescence on transversal section of a mouse heart transgenic for the M10 enhancer showing the colocalization of the ß-galactosidase with HCN4 in the SAN. RA, right atria. TV, tricuspid valve; RVV, right venous valve, RAVJ, right atrioventricular junction; RSCV, right superior cava vein; SAN, sinoatrial node; AVN, atrioventricular node.
(TIF)

S5 Fig Description of the affected area of the AV junction in *Furin* mutant hearts. (A-E’) Images of Trichrome stained sections of an adult Furin mutant heart. A’ to B’ represent high magnifications of the black squared areas in A to E. (F) Schematic representation of the AV junction, top view. The positions of the sections are indicated with dashed lines. AVN, AV node. The blue area in the lateral side of the Left AV junction indicates the affected area.
(TIF)

S6 Fig Uncropped images of Western blots and EMSA presented in the manuscript. (A) EMSA from Fig 1C and S1C Fig. (B-D) Western Blots from Fig 3Q. Dashed red square show where images were cropped for publication.
(TIF)

S1 Table Summary of ultrasound parameters recorded in three months old mutant mice. (DOCX)

## References

[1] Prall OW, Menon MK, Solloway MJ, Watanabe Y, Zaffran S, Bajolle F, et al An Nkx2-5/Bmp2/Smad1 negative feedback loop controls heart progenitor specification and proliferation. Cell. 2007;128(5):947–59. Epub 2007/03/14. S0092-8674(07)00243-7 [pii] 10.1016/j.cell.2007.01.042 17350578 PMC2092439

[2] Akazawa H, Komuro I. Cardiac transcription factor Csx/Nkx2-5: Its role in cardiac development and diseases. Pharmacol Ther. 2005;107(2):252–68. Epub 2005/06/01. S0163-7258(05)00065-3 [pii] 10.1016/j.pharmthera.2005.03.005 . 15925411

[3] Nakashima Y, Yanez DA, Touma M, Nakano H, Jaroszewicz A, Jordan MC, et al Nkx2-5 suppresses the proliferation of atrial myocytes and conduction system. Circ Res. 2014;114(7):1103–13. Epub 2014/02/25. CIRCRESAHA.114.303219 [pii] 10.1161/CIRCRESAHA.114.303219 . 24563458

[4] Briggs LE, Takeda M, Cuadra AE, Wakimoto H, Marks MH, Walker AJ, et al Perinatal loss of Nkx2-5 results in rapid conduction and contraction defects. Circ Res. 2008;103(6):580–90. Epub 2008/08/12. CIRCRESAHA.108.171835 [pii] 10.1161/CIRCRESAHA.108.171835 18689573 PMC2590500

[5] Jay PY, Harris BS, Maguire CT, Buerger A, Wakimoto H, Tanaka M, et al Nkx2-5 mutation causes anatomic hypoplasia of the cardiac conduction system. J Clin Invest. 2004;113(8):1130–7. Epub 2004/04/16. 10.1172/JCI19846 15085192 PMC385399

[6] Tanaka M, Chen Z, Bartunkova S, Yamasaki N, Izumo S. The cardiac homeobox gene Csx/Nkx2.5 lies genetically upstream of multiple genes essential for heart development. Development. 1999;126(6):1269–80. Epub 1999/02/18. 10021345 10.1242/dev.126.6.1269

[7] Dupays L, Jarry-Guichard T, Mazurais D, Calmels T, Izumo S, Gros D, et al Dysregulation of connexins and inactivation of NFATc1 in the cardiovascular system of Nkx2-5 null mutants. J Mol Cell Cardiol. 2005;38(5):787–98. Epub 2005/04/27. S0022-2828(05)00064-7 [pii] 10.1016/j.yjmcc.2005.02.021 . 15850572

[8] Christoffels VM, Smits GJ, Kispert A, Moorman AF. Development of the pacemaker tissues of the heart. Circ Res. 2010;106(2):240–54. Epub 2010/02/06. 106/2/240 [pii] 10.1161/CIRCRESAHA.109.205419 . 20133910

[9] Thomas G. Furin at the cutting edge: from protein traffic to embryogenesis and disease. Nat Rev Mol Cell Biol. 2002;3(10):753–66. 10.1038/nrm934 . 12360192 PMC1964754

[10] Cui Y, Hackenmiller R, Berg L, Jean F, Nakayama T, Thomas G, et al The activity and signaling range of mature BMP-4 is regulated by sequential cleavage at two sites within the prodomain of the precursor. Genes Dev. 2001;15(21):2797–802. 10.1101/gad.940001 . 11691831 PMC312809

[11] Logeat F, Bessia C, Brou C, LeBail O, Jarriault S, Seidah NG, et al The Notch1 receptor is cleaved constitutively by a furin-like convertase. Proc Natl Acad Sci USA. 1998;95(14):8108–12. . 9653148 10.1073/pnas.95.14.8108 PMC20937

[12] Bessonnard S, Mesnard D, Constam DB. PC7 and the related proteases Furin and Pace4 regulate E-cadherin function during blastocyst formation. J Cell Biol. 2015;210(7):1185–97. Epub 2015/09/30. jcb.201503042 [pii] 10.1083/jcb.201503042 . 26416966 PMC4586756

[13] Cui Y, Jean F, Thomas G, Christian JL. BMP-4 is proteolytically activated by furin and/or PC6 during vertebrate embryonic development. EMBO J. 1998;17(16):4735–43. 10.1093/emboj/17.16.4735 . 9707432 PMC1170802

[14] Jiao K, Kulessa H, Tompkins K, Zhou Y, Batts L, Baldwin H, et al An essential role of Bmp4 in the atrioventricular septation of the mouse heart. Genes Dev. 2003;17(19):2362–7. 10.1101/gad.1124803 . 12975322 PMC218073

[15] Goldman D, Hackenmiller R, Nakayama T, Sopory S, Wong C, Kulessa H, et al Mutation of an upstream cleavage site in the BMP4 prodomain leads to tissue-specific loss of activity. Development (Cambridge, England). 2006;133(10):1933–42. 10.1242/dev.02368 . 16624858

[16] Roebroek A, Taylor N, Louagie E, Pauli I, Smeijers L, Snellinx A, et al Limited redundancy of the proprotein convertase furin in mouse liver. J Biol Chem. 2004;279(51):53442–50. 10.1074/jbc.M407152200 . 15471862

[17] Kothary R, Clapoff S, Darling S, Perry MD, Moran LA, Rossant J. Inducible expression of an hsp68-lacZ hybrid gene in transgenic mice. Development. 1989;105(4):707–14. . 2557196 10.1242/dev.105.4.707

[18] Ran FA, Hsu PD, Wright J, Agarwala V, Scott DA, Zhang F. Genome engineering using the CRISPR-Cas9 system. Nat Protoc. 2013;8(11):2281–308. 10.1038/nprot.2013.143 24157548 PMC3969860

[19] Roberts TA, Price AN, Jackson LH, Taylor V, David AL, Lythgoe MF, et al Direct comparison of high-temporal-resolution CINE MRI with Doppler ultrasound for assessment of diastolic dysfunction in mice. NMR Biomed. 2017;30(10). Epub 2017/06/24. 10.1002/nbm.3763 28643891 PMC5638074

[20] Dupays L, Kotecha S, Angst B, Mohun TJ. Tbx2 misexpression impairs deployment of second heart field derived progenitor cells to the arterial pole of the embryonic heart. Dev Biol. 2009;333(1):121–31. Epub 2009/07/01. S0012-1606(09)00994-4 [pii] 10.1016/j.ydbio.2009.06.025 . 19563797

[21] Mohun TJ, Weninger WJ. Imaging heart development using high-resolution episcopic microscopy. Curr Opin Genet Dev. 2011;21(5):573–8. Epub 2011/09/07. S0959-437X(11)00111-0 [pii] 10.1016/j.gde.2011.07.004 . 21893408 PMC3368266

[22] Dupays L, Shang C, Wilson R, Kotecha S, Wood S, Towers N, et al Sequential Binding of MEIS1 and NKX2-5 on the Popdc2 Gene: A Mechanism for Spatiotemporal Regulation of Enhancers during Cardiogenesis. Cell Rep. 2015;13(1):183–95. Epub 2015/09/29. 10.1016/j.celrep.2015.08.065 26411676 PMC4597108

[23] Dupays L, Mohun T. Spatiotemporal regulation of enhancers during cardiogenesis. Cell Mol Life Sci. 2017;74(2):257–65. Epub 2016/08/09. 10.1007/s00018-016-2322-y 27497925 PMC5219004

[24] Roebroek A, Umans L, Pauli I, Robertson E, van Leuven F, Van de Ven W, et al Failure of ventral closure and axial rotation in embryos lacking the proprotein convertase Furin. Development. 1998;125(24):4863–76. . 9811571 10.1242/dev.125.24.4863

[25] Cai C-L, Martin JC, Sun Y, Cui L, Wang L, Ouyang K, et al A myocardial lineage derives from Tbx18 epicardial cells. Nature. 2008;454(7200):104–8. 10.1038/nature06969 18480752 PMC5540369

[26] Stanley EG, Biben C, Elefanty A, Barnett L, Koentgen F, Robb L, et al Efficient Cre-mediated deletion in cardiac progenitor cells conferred by a 3'UTR-ires-Cre allele of the homeobox gene Nkx2-5. Int J Dev Biol. 2002;46(4):431–9. Epub 2002/07/27. . 12141429

[27] Breckenridge R, Kotecha S, Towers N, Bennett M, Mohun T. Pan-myocardial expression of Cre recombinase throughout mouse development. Genesis. 2007;45(3):135–44. Epub 2007/03/06. 10.1002/dvg.20275 . 17334998

[28] Hoogaars WM, Tessari A, Moorman AF, De Boer PA, Hagoort J, Soufan AT, et al The transcriptional repressor Tbx3 delineates the developing central conduction system of the heart. Cardiovasc Res. 2004;62(3):489–99. 10.1016/j.cardiores.2004.01.030 . 15158141

[29] Liang X, Wang G, Lin L, Lowe J, Zhang Q, Bu L, et al HCN4 dynamically marks the first heart field and conduction system precursors. Circ Res. 2013;113(4):399–407. Epub 2013/06/08. 10.1161/CIRCRESAHA.113.301588 23743334 PMC4017870

[30] Kruithof BP, Krawitz SA, Gaussin V. Atrioventricular valve development during late embryonic and postnatal stages involves condensation and extracellular matrix remodeling. Dev Biol. 2007;302(1):208–17. Epub 2006/10/24. 10.1016/j.ydbio.2006.09.024 . 17054936

[31] Gaussin V, Morley G, Cox L, Zwijsen A, Vance K, Emile L, et al Alk3/Bmpr1a receptor is required for development of the atrioventricular canal into valves and annulus fibrosus. Circulation research. 2005;97(3):219–26. 10.1161/01.RES.0000177862.85474.63 . 16037571 PMC2950023

[32] Stroud DM, Gaussin V, Burch JBE, Yu C, Mishina Y, Schneider MD, et al Abnormal conduction and morphology in the atrioventricular node of mice with atrioventricular canal targeted deletion of Alk3/Bmpr1a receptor. Circulation. 2007;116(22):2535–43. 10.1161/CIRCULATIONAHA.107.696583 . 17998461 PMC2947829

[33] Osterwalder M, Barozzi I, Tissieres V, Fukuda-Yuzawa Y, Mannion BJ, Afzal SY, et al Enhancer redundancy provides phenotypic robustness in mammalian development. Nature. 2018;554(7691):239–43. Epub 2018/02/09. 10.1038/nature25461 29420474 PMC5808607

[34] Kim W, Essalmani R, Szumska D, Creemers JW, Roebroek AJ, D'Orleans-Juste P, et al Loss of endothelial furin leads to cardiac malformation and early postnatal death. Mol Cell Biol. 2012;32(17):3382–91. Epub 2012/06/27. MCB.06331-11 [pii] 10.1128/MCB.06331-11 . 22733989 PMC3422005

[35] Constam DB, Robertson EJ. Tissue-specific requirements for the proprotein convertase furin/SPC1 during embryonic turning and heart looping. Development. 2000;127(2):245–54. . 10603343 10.1242/dev.127.2.245

[36] Francou A, De Bono C, Kelly RG. Epithelial tension in the second heart field promotes mouse heart tube elongation. Nat Commun. 2017;8:14770 Epub 2017/03/31. 10.1038/ncomms14770 28357999 PMC5379109

[37] Tirosh-Finkel L, Zeisel A, Brodt-Ivenshitz M, Shamai A, Yao Z, Seger R, et al BMP-mediated inhibition of FGF signaling promotes cardiomyocyte differentiation of anterior heart field progenitors. Development. 2010;137(18):2989–3000. Epub 2010/08/13. 10.1242/dev.051649 . 20702560

[38] McCulley DJ, Kang JO, Martin JF, Black BL. BMP4 is required in the anterior heart field and its derivatives for endocardial cushion remodeling, outflow tract septation, and semilunar valve development. Dev Dyn. 2008;237(11):3200–9. Epub 2008/10/17. 10.1002/dvdy.21743 18924235 PMC2728547

[39] Nelsen SM, Christian JL. Site-specific cleavage of BMP4 by furin, PC6, and PC7. J Biol Chem. 2009;284(40):27157–66. Epub 2009/08/05. 10.1074/jbc.M109.028506 19651771 PMC2785643

[40] Danesh SM, Villasenor A, Chong D, Soukup C, Cleaver O. BMP and BMP receptor expression during murine organogenesis. Gene Expr Patterns. 2009;9(5):255–65. Epub 2009/04/28. 10.1016/j.gep.2009.04.002 19393343 PMC2709213

[41] Ashraf H, Pradhan L, Chang EI, Terada R, Ryan NJ, Briggs LE, et al A mouse model of human congenital heart disease: high incidence of diverse cardiac anomalies and ventricular noncompaction produced by heterozygous Nkx2-5 homeodomain missense mutation. Circ Cardiovasc Genet. 2014;7(4):423–33. Epub 2014/07/17. CIRCGENETICS.113.000281 [pii] 10.1161/CIRCGENETICS.113.000281 . 25028484 PMC4140955

[42] Chowdhury R, Ashraf H, Melanson M, Tanada Y, Nguyen M, Silberbach M, et al Mouse Model of Human Congenital Heart Disease: Progressive Atrioventricular Block Induced by a Heterozygous Nkx2-5 Homeodomain Missense Mutation. Circ Arrhythm Electrophysiol. 2015;8(5):1255–64. Epub 2015/08/01. CIRCEP.115.002720 [pii] 10.1161/CIRCEP.115.002720 . 26226998 PMC4618020

[43] Schott JJ, Benson DW, Basson CT, Pease W, Silberbach GM, Moak JP, et al Congenital heart disease caused by mutations in the transcription factor NKX2-5. Science. 1998;281(5373):108–11. Epub 1998/07/04. . 9651244 10.1126/science.281.5373.108

[44] Reamon-Buettner SM, Borlak J. NKX2-5: an update on this hypermutable homeodomain protein and its role in human congenital heart disease (CHD). Hum Mutat. 2010;31(11):1185–94. Epub 2010/08/21. 10.1002/humu.21345 . 20725931

[45] Pfeufer A, van Noord C, Marciante KD, Arking DE, Larson MG, Smith AV, et al Genome-wide association study of PR interval. Nat Genet. 2010;42(2):153–9. Epub 2010/01/12. ng.517 [pii] 10.1038/ng.517 20062060 PMC2850197

[46] Kasahara H, Wakimoto H, Liu M, Maguire CT, Converso KL, Shioi T, et al Progressive atrioventricular conduction defects and heart failure in mice expressing a mutant Csx/Nkx2.5 homeoprotein. J Clin Invest. 2001;108(2):189–201. Epub 2001/07/18. 10.1172/JCI12694 11457872 PMC203028

[47] Aanhaanen WT, Boukens BJ, Sizarov A, Wakker V, de Gier-de Vries C, van Ginneken AC, et al Defective Tbx2-dependent patterning of the atrioventricular canal myocardium causes accessory pathway formation in mice. J Clin Invest. 2011;121(2):534–44. Epub 2011/01/27. 44350 [pii] 10.1172/JCI44350 . 21266775 PMC3026729

[48] Rentschler S, Harris BS, Kuznekoff L, Jain R, Manderfield L, Lu MM, et al Notch signaling regulates murine atrioventricular conduction and the formation of accessory pathways. J Clin Invest. 2011;121(2):525–33. Epub 2011/01/27. 44470 [pii] 10.1172/JCI44470 . 21266778 PMC3026731

[49] Gillers BS, Chiplunkar A, Aly H, Valenta T, Basler K, Christoffels VM, et al Canonical wnt signaling regulates atrioventricular junction programming and electrophysiological properties. Circ Res. 2015;116(3):398–406. Epub 2015/01/20. CIRCRESAHA.116.304731 [pii] 10.1161/CIRCRESAHA.116.304731 . 25599332 PMC4312529

