# Supplementary figures and images for: Furin, a transcriptional target of NKX2-5, has an essential role in heart development and function
Source: PLoS One. 2019 Mar 6;14(3):e0212992. doi: 10.1371/journal.pone.0212992 (PMC6402701; doi:10.1371/journal.pone.0212992)

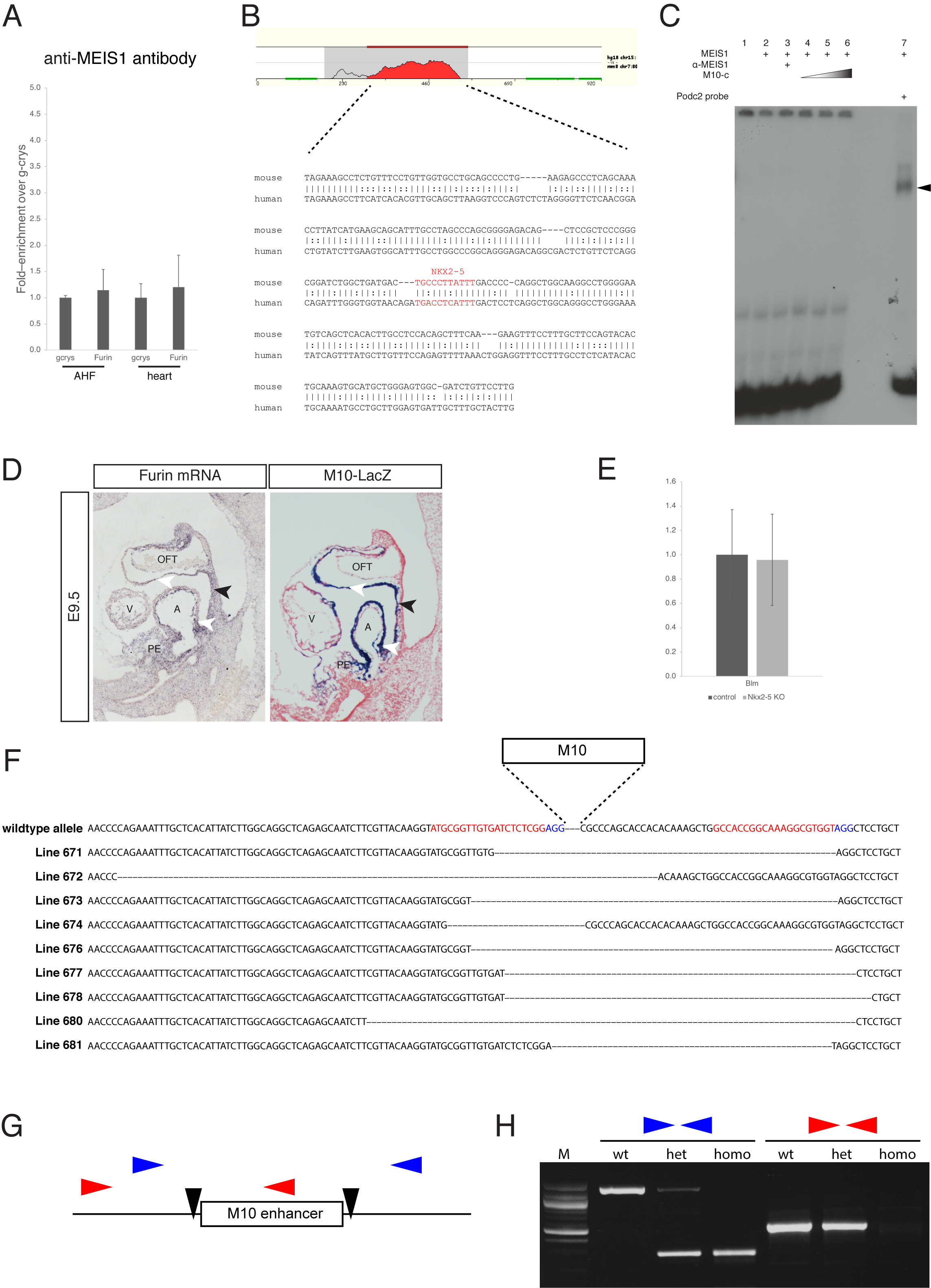

Supplement: S1 Fig — (A) ChIP analysis using chromatin purified from E9.5 AHF and heart and an anti-MEIS1 antibody. Note that there is no significant difference in enrichment of binding between Furin and a negative control region derived from the gamma-crystallin gene. Representative results from quantitative PCR using primers for the M10 region are presented. (B) Diagram showing the global conservation of the M10 sequence (920bp) between mouse (mm8) and human (hg18). The area in red (269bp in mouse) is highly conserved (sequence conservation superior at 70%). A base level sequence comparison is shown underneath. The position of the Nkx2-5 DNA binding site analysed in that study is shown in red. (C) EMSA showing that MEIS1 doesn’t bind the M10 probe in vitro (Lanes 2 to lane 6) while it binds the Popdc2 enhancer identified previously (Lane 7, black arrowhead). (D) Sagittal sections of embryos at E9.5. White arrowheads indicate expression in the myocardium while black arrowheads indicate expression in the anterior heart field. PE, proepicardium. (E) Relative quantitative RT-PCR for Blm mRNA expression in isolated embryo hearts of control and Nkx2-5 knock-out (Nkx2-5-gfp/Nkx2-5-gfp) at E9.5. (F) Sequence of the allele deleted in the 9 stable lines generated (Line 671-Line 681). Lines 676 and Line 678 were kept for subsequent analysis. sgRNA sequences are labelled in red, PAM sequences in blue. (G) Diagram showing the strategy used for offsprings genotyping. The schematic position of the sgRNA used to ablate the M10 enhancer in the genome is indicated with black arrowheads. The internal (red arrowheads) and overlapping (blue arrowheads) primer pairs used for genotyping are shown. (H) PCR analysis of the DNA of three mice (wildtype, heterozygote and homozygote for the deletion). Primers used in each lane are indicated with coloured arrowheads. Note the absence of amplification with the red/internal primer in the homozygous line. (TIF) [file pone.0212992.s001.tif]

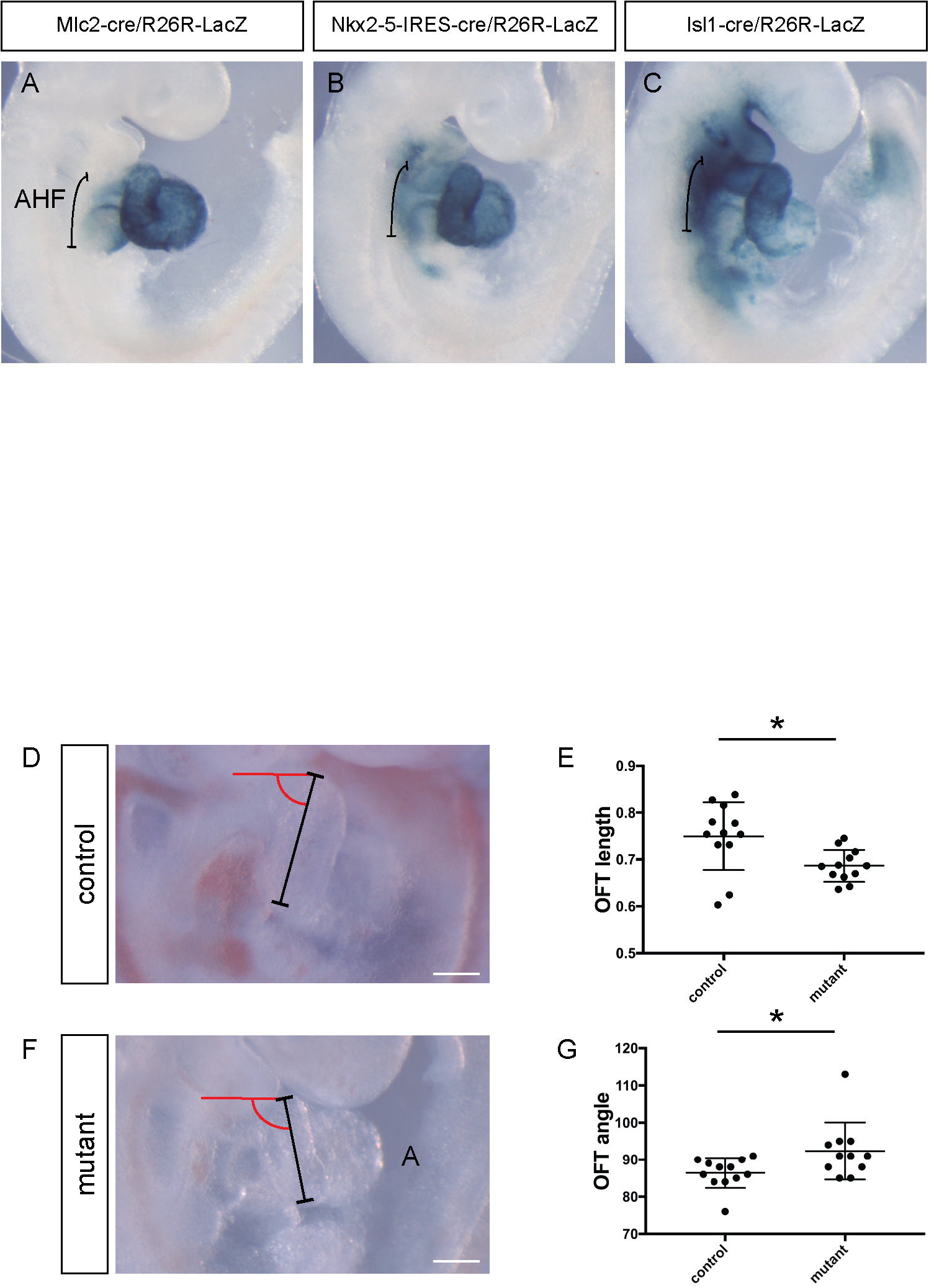

Supplement: S2 Fig — (A-C) At E9.5 Isl1cre/+ and Nkx2-5IRES-Cre/+ lines are expressed in the AHF while Mlc2-cre line is not (Brackets). Expression of the reporter LacZ appears to be broader and stronger in the AHF of the Isl1-cre/R26R-LacZ embryos compare to Nkx2-5-IRES-cre/R26R-LacZ embryos (Compare brackets in B and C). (D, F) Right side views of E9.5 control and Isl1cre/+/Furinfl/fl mutant embryos. (E) Measurement of OFT length as shown in D and F with black bars. (F) Measurement of OFT angle as shown in D and F in red (n = 12 for control embryos and 12 for Furin mutants). *P<0.05 for unpaired Student’s t test. (TIF) [file pone.0212992.s002.tif]

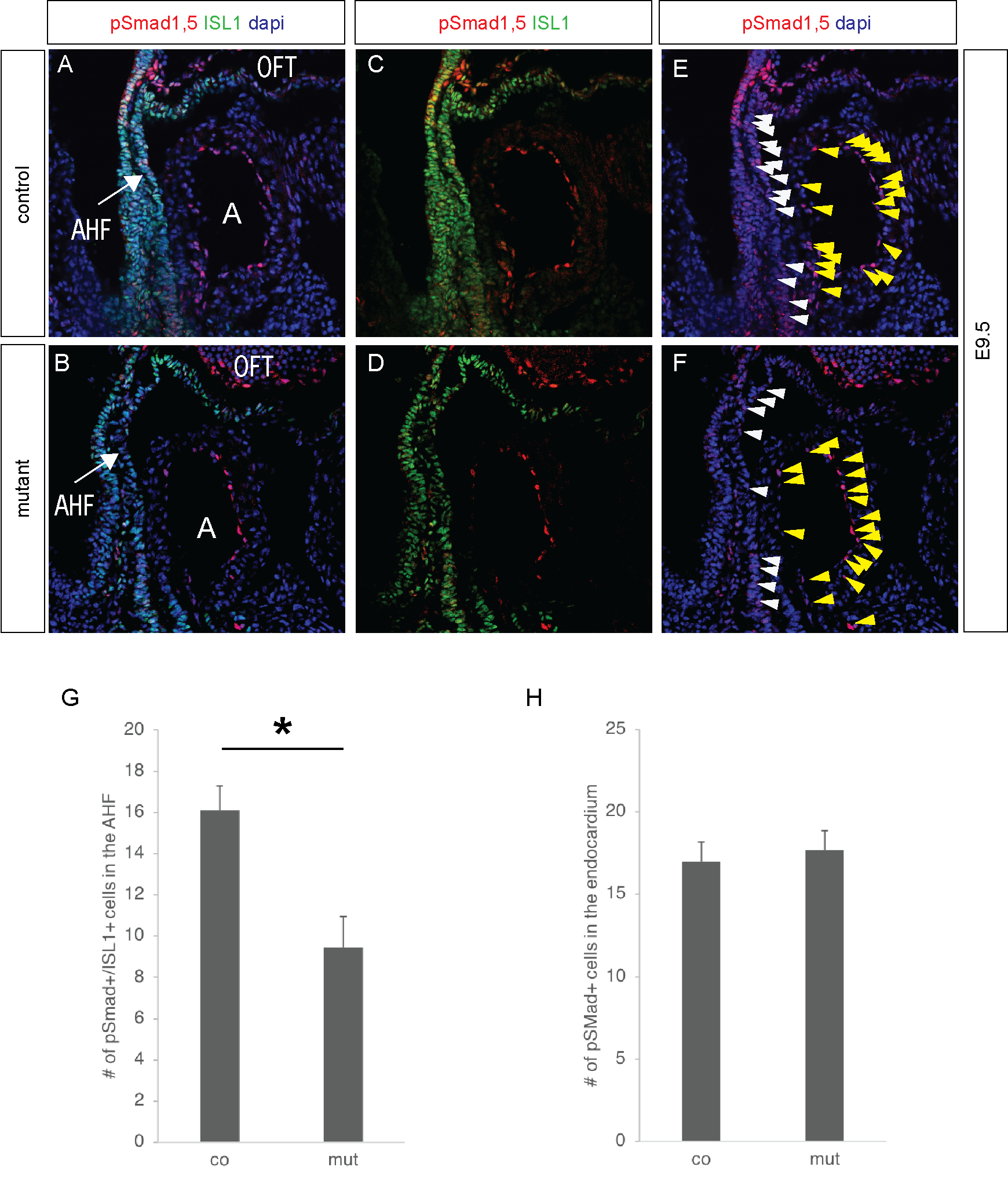

Supplement: S3 Fig — (A-F) Immunostaining showing the expression of pSMAD1/5 (red) and ISL1 (green) in a wildtype (A, C and E) and mutant (B, D and F) embryo E9.5 mouse heart on sagittal sections. White arrowheads indicate phospho-SMAD1/5 positive / ISL1 positive cells in the AHF. Yellow arrowheads indicate phospho-SMAD1/5 positive endocardial cells of the atria. (G) Graph showing a significant reduction in the number of phospho-SMAD1/5 / ISL1 positive cells in the AHF. *P<0.05 for unpaired Student’s t test. (H) Graph showing that the number of phospho-SMAD1/5 positive cells in the endocardium of the atria in wildtype (n = 3) and control (n = 3) is not significantly changed. (TIF) [file pone.0212992.s003.tif]

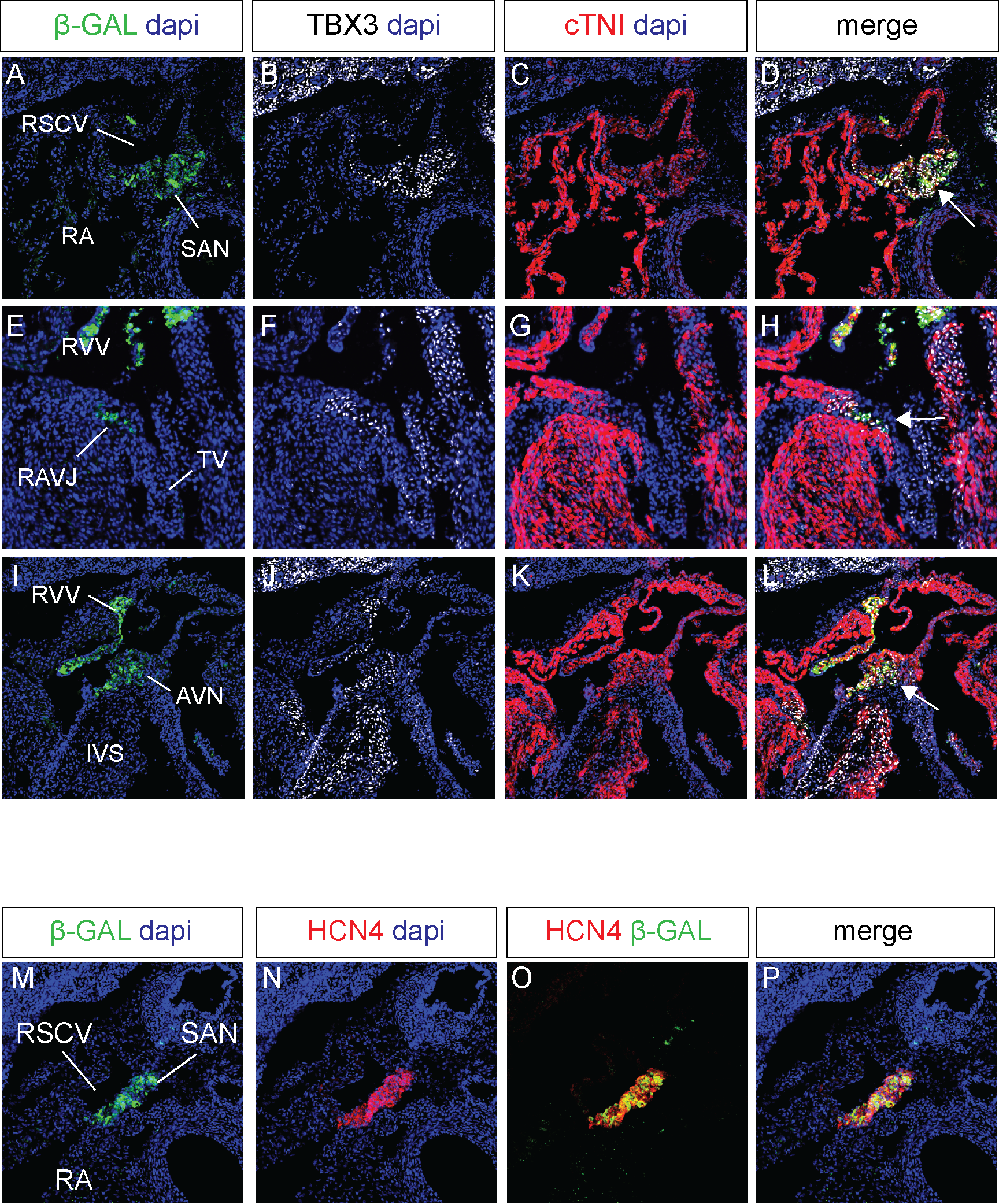

Supplement: S4 Fig — Expression of the transgene M10 is detected with an antibody against the ß-galactosidase. Panels show transversal section of a E17.5 mouse heart with a focus on the sinoatrial node (A-D), the tricuspid valve (E-H) and AVN (I-L). White arrows in D, H and L show that the transgene M10 is expressed in the sinoatrial node (D), the right atrioventricular junction (H) and the atrioventricular node (L). (M-P) Immunofluorescence on transversal section of a mouse heart transgenic for the M10 enhancer showing the colocalization of the ß-galactosidase with HCN4 in the SAN. RA, right atria. TV, tricuspid valve; RVV, right venous valve, RAVJ, right atrioventricular junction; RSCV, right superior cava vein; SAN, sinoatrial node; AVN, atrioventricular node. (TIF) [file pone.0212992.s004.tif]

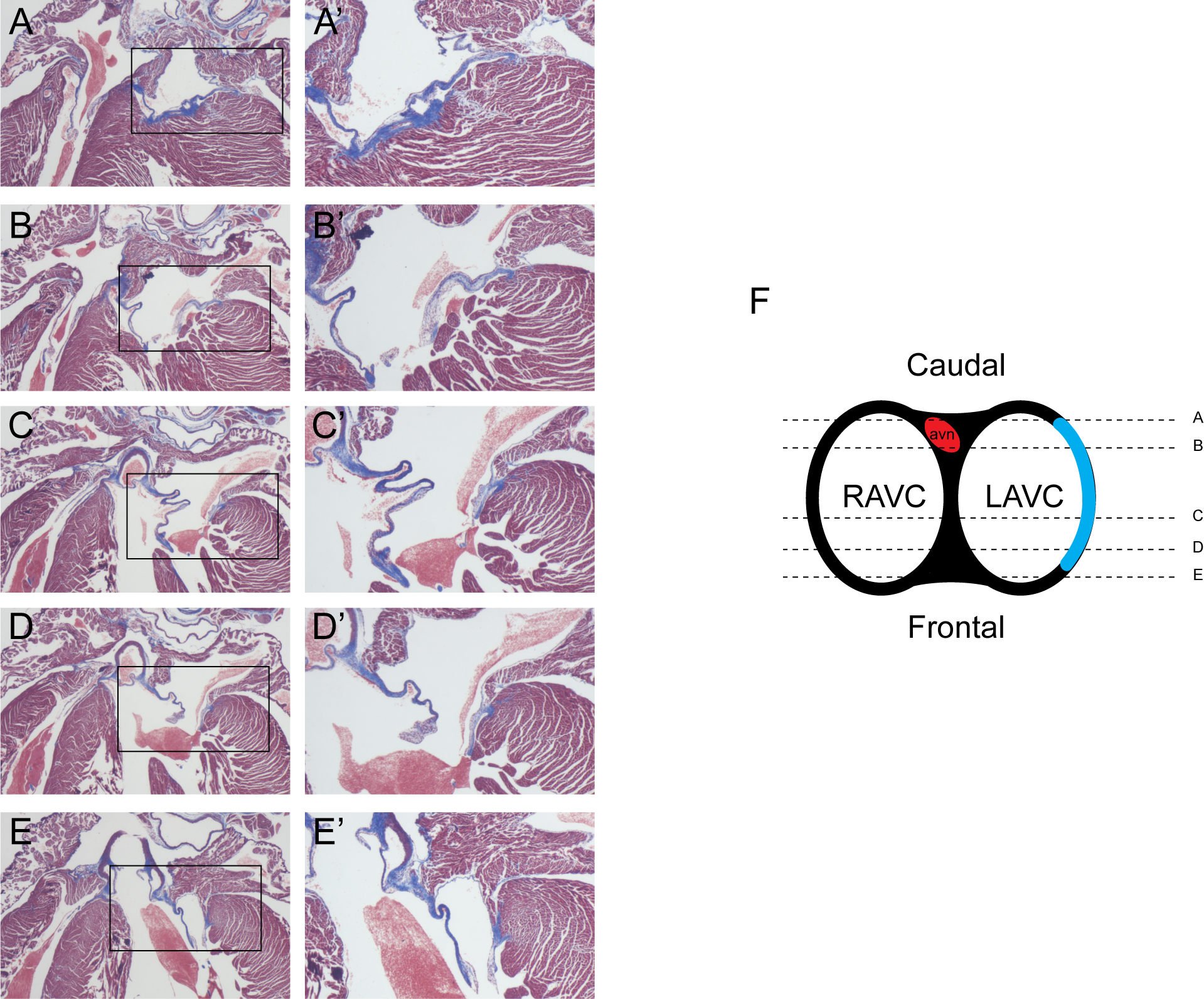

Supplement: S5 Fig — (A-E’) Images of Trichrome stained sections of an adult Furin mutant heart. A’ to B’ represent high magnifications of the black squared areas in A to E. (F) Schematic representation of the AV junction, top view. The positions of the sections are indicated with dashed lines. AVN, AV node. The blue area in the lateral side of the Left AV junction indicates the affected area. (TIF) [file pone.0212992.s005.tif]

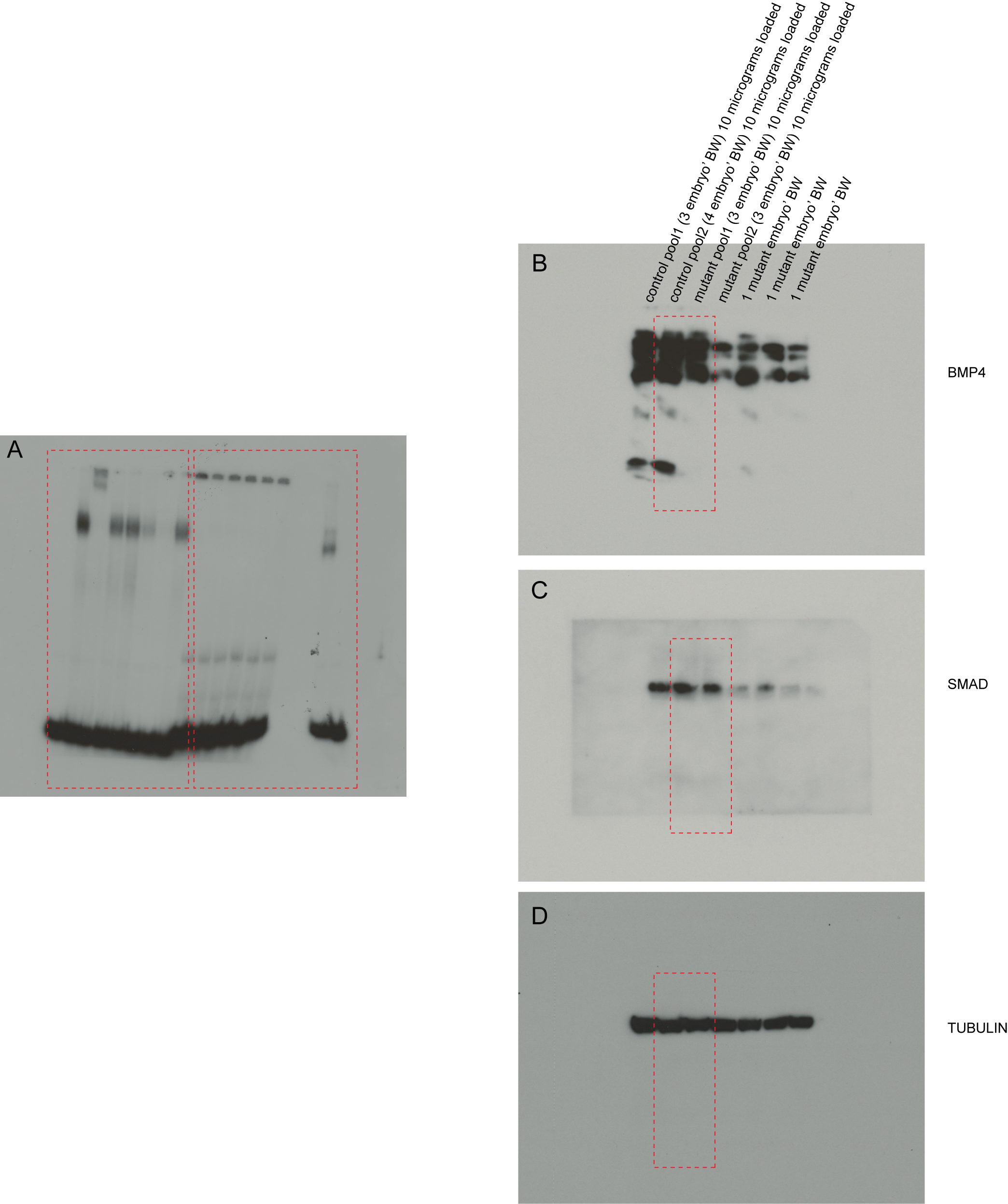

Supplement: S6 Fig — (A) EMSA from Fig 1C and S1C Fig. (B-D) Western Blots from Fig 3Q. Dashed red square show where images were cropped for publication. (TIF) [file pone.0212992.s006.tif]
